# Ligand‐Driven Annular‐Epitaxial Growth of CuS‐Au Heterostructures as Trinity Plasmonic Nanozyme for Multimode Diagnosis of Pathogenic Bacteria

**DOI:** 10.1002/advs.202500134

**Published:** 2025-04-24

**Authors:** Xiaorui Lin, Pengyou Zhou, Miao An, Chenyi Zhu, Yuanfeng Pang, Rui Xiao

**Affiliations:** ^1^ Capital Medical University No.10 Xitoutiao, You An Men Beijing 100069 China; ^2^ State Key Laboratory of Pathogen and Biosecurity Academy of Military Medical Sciences Beijing 100071 China

**Keywords:** annular‐epitaxial growth, CuS‐Au heterostructures, ligand‐driven, multimode diagnosis of pathogenic bacteria, trinity plasmonic nanozyme

## Abstract

This study presents a novel method to control the site‐selective growth of Au nanostars on CuS nanodisc substrate, it indicates that the surfactant ligands play a key role in the architecture control, only CTAC and homologous series with appropriate affinity to CuS can direct the annular‐epitaxial growth of Au nanoparticles on the CuS, which demonstrates superior peroxidase (POD)‐mimic and SERS activity. Mechanistic studies indicate that plasmon‐enhanced catalytic and SERS activity can be attributed to the spatially separated CuS‐Au heterostructure, which supports the light‐triggered hot electron‐hole pairs production and localized surface plasmon resonance hotspots. For practical biosensing, the CuS‐Au heterostructures assembled lateral flow assay (LFA) was used for SERS/catalytic colorimetric/photothermal three‐mode detection of *Streptococcus pneumoniae* and *Klebsiella pneumoniae*, with visually colorimetric mode at 103 CFU/mL and quantitative SERS/photothermal modes at 2–10^2^ CFU/mL within 15 min, 15 clinical samples were used to validate the assay, the result was 100% concordant to the results of quantitative real‐time PCR. This study provides a unique avenue to controllably produce plasmon‐enhanced nanozyme, which can provide multi‐mode signals for LFA application and meet the requirements of different scenarios.

## Graphical abstract

CuS‐Au heterostructures were synthesized via ligand‐regulated annular‐epitaxial growth strategy, biotin‐Concanavalin A (ConA) was modified on the CuS‐Au heterostructures for universal bacterial‐labelling, assembled with strip, *Streptococcus pneumoniae* and *Klebsiella pneumoniae* can be sensitively recognized by SERS/colorimetric/temperature three‐mode signals within 20 min simultaneously.

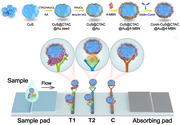

## 1 Introduction

Bacterial infections have become a critical global health challenge and caused almost 6.7 million annual deaths.^[^ ^1^ ^]^ Among of them, *Streptococcus pneumoniae* (*S. pneumoniae*) and *Klebsiella pneumoniae* (*K. pneumoniae*) emerge as major contributors to the substantial morbidity through community‐acquired and hospital‐acquired pneumonias respectively. These pathogens frequently lead to severe complications including sepsis and multiorgan failure, especially for elderly and immunocompromised populations.^[^ ^2^, ^3^ ^]^ Conventional culture‐based methods require prolonged processing times (24 hours to several days) and complicated operation, molecular diagnosis methods such as PCR and gene sequencing can offer faster and sensitive detection, however, they require expensive instruments, complex nucleic acid amplification steps, and skilled technicians, which limits the point‐of‐care testing (POCT) especially in resource‐limited areas.^[^ ^4^ ^]^ Therefore, it is urgently needed to develop simple and sensitive strategy for synchronous recognition of *S. pneumoniae* and *K. pneumonia* in biosamples.

Lateral flow assay (LFA) offers core advantages such as rapid detection, simple operation, no requirement for complex instruments, and visually interpretable results, making it suitable for POCT scenarios. However, current commercially available colloidal gold‐based lateral flow strips typically rely on single colloidal gold labeling, resulting in insufficient sensitivity and dependence on semi‐quantitative visual interpretation, which hampers accurate detection of low‐abundance targets.^[^ ^5^ ^]^ Therefore, developing highly sensitive LFA systems has become a key research focus.

Nanozyme is a type of nanomaterial inherently endowed with enzyme‐mimic characteristics,^[^ ^6^ ^]^ compared with natural enzymes, nanozyme possesses many advantages such as high stability, multi‐enzyme simulation activity, unique surface chemical properties, and biocompatibility.^[^ ^7^, ^8^, ^9^ ^]^ Since first reported in 2007,^[^ ^10^ ^]^ a series of nanozymes such as peroxidase (POD)‐mimic nanozymes, oxidase (OXD)‐mimic nanozymes, catalase (CAT)‐mimic nanozymes and laccase‐mimic nanozymes have been used in the development of biosensing systems, providing new ideas for the point‐of‐care testing (POCT) technologies.^[^ ^11^, ^12^, ^13^ ^]^ Among them, POD‐mimic nanozymes are the most commonly used catalyzed colorimetric tags as the mimetic POD activity can catalyze 3, 3′, 5, 5′‐tetramethylbenzidine (TMB) to blue oxidized TMB (ox‐TMB) with enhanced colorimetric signals.^[^ ^14^ ^]^ However, most of the nanozymes based biosensors depend on semi‐quantitative colorimetric signal analysis mode, which can limit the accuracy and sensitivity of the biosensors.^[^ ^15^, ^16^ ^]^ Therefore, it is of great desire to explore the multi‐functional signal ability of the individual nanozymes.

Surface‐enhanced Raman scattering (SERS) is a powerful analytical technique with advantages of high sensitivity, anti‐interference, and precise quantitative ability, SERS can recognize trace targets especially in complex bio‐samples.^[^ ^17^, ^18^ ^]^ The development of nanozymes endowed with SERS activity can be a superior strategy to enhance the sensitivity and quantitative ability in multi‐scenario detection application.^[^ ^19^, ^20^ ^]^ Recently, several plasmonic nanozymes^[^ ^21^, ^22^, ^23^ ^]^ have been reported for SERS and catalytic colorimetric detections. Although displaying dual‐mode signals, most of them depended on the simple combination of SERS constituents Au and nanozyme constituents such as Pt, typically, the SERS Au substrate was covered by nanozyme metal shell or nanozyme small particles.^[^ ^24^, ^25^, ^26^ ^]^ Actually, according to previous researches, the SERS activity and POD activity of the individual nanoparticle can be a pair of paradoxes,^[^ ^27^, ^28^ ^]^ even a small amount of Pt deposition can cause a significant SERS activity decreasing for the Au or Ag SERS substrates^[^ ^29^, ^30^, ^31^ ^]^, thus it is important to develop a controllable method to obtain effective SERS nanozymes.

Plasmon‐enhanced nanozyme is a type of plasmonic nanoparticle, for which, elevated enzyme‐mimicking activity can be obtained by light irradiation inducing localized surface plasmon resonance (LSPR).^[^ ^32^ ^]^ Recently, methods for precise controlling of the structure of plasmonic nanozyme have been reported,^[^ ^33^ ^]^ for example, Au nanobipyramids or Au nanorods based dumbbell‐shaped Au‐Pt nanostructures have been reported for plasmon‐enhanced POD‐mimic activity of nanozymes,^[^ ^22^, ^33^ ^]^ according to the controllable distribution of Pt on the Au nanobipyramids or nanorods to form heterostructures, better plasmon‐enhanced catalytic activity can be obtained.^[^ ^22^, ^34^ ^]^ However, the SERS activity of those plasmon‐enhanced nanozyme heterostructures was rarely discussed. On the other hand, the method for controllable selective growth of plasmonic metal on transition metals based nanozyme substrate to obtain SERS‐nanozyme dual activity heterostructures has never been researched.

Herein, we developed a simple ligand‐regulation strategy for the controllable annular‐epitaxial growth of Au nanoparticles on the surface of CuS nanodisc. CuS nanodisc was a typical 2D semiconductor nanomaterial with superior POD activity,^[^ ^35^ ^]^ compared with commonly used noble metals nanozymes such as Pt, Pd, CuS nanodisc displays higher POD activity, better stability and lower cost.^[^ ^36^ ^]^ Several CuS based nanozyme biosensors have been reported,^[^ ^35^, ^36^, ^37^, ^38^ ^]^ however, the controllable strategy to obtain plasmonic CuS based heterostructures with both of SERS and nanozyme dual activities has never been reported. In this research, various surfactant ligands cetyltrimethylammonium bromide (CTAB), Cetyltrimethylammonium chloride (CATC), polyvinyl pyrrolidone (PVP) were first used to respectively modify the surface of CuS nanodisc, results indicated that only CTAC can control the annular‐epitaxial growth of Au nanoparticles on the CuS substrate, inducing both of superior POD and SERS activity. For both CTAB or PVP ligands, Au nanoparticles distributed on the CuS substrate individually or carpetedly, inducing weak SERS or catalytic activity. Ulteriorly, the regulated site‐selective growth of Au depended on CTAC packing density, only under middle CTAC concentrations, the annular‐epitaxial CuS‐Au heterostructure can be obtained. Moreover, the obtained annular‐epitaxial CuS‐Au heterostructure exhibited an excellent near‐infrared (NIR) photothermal performance. Therefore, SERS, catalytic colorimetric, and photothermal multimode signals can be obtained attributed to the CTAC regulated CuS‐Au heterostructure, which can be used as single‐component and three‐functional signal probe with visual and precisely quantitative ability in the multi‐scenario sensing.

Based on the above‐mentioned characters, CTAC regulated CuS‐Au heterostructure was assembled with lateral flow assay (LFA) for SERS/colorimetric/photothermal multimode visual/quantitative detection of *S. pneumoniae* and *K. pneumoniae*. *S. pneumoniae* and *K. pneumoniae* are two of the most common pathogenic bacteria to induce lung infections.^[^ ^1^, ^39^, ^40^ ^]^ However, until now, no rapid LFA method for simultaneous diagnosis of *K. pneumoniae* and *S. pneumoniae* has been reported. Herein, for the first time, we constructed CuS‐Au based multimode LFA for *S. pneumoniae* and *K. pneumoniae* synchronously rapid detection. We analyzed the detection range and sensitivity of the proposed three‐mode LFA sensing platforms. The colorimetric mode can be used as rapid POCT tool for pathogenic bacterium diagnosis with a detection limit of 1.2 × 10^3^ CFU/mL, the SERS and photothermal modes can be used as ultrasensitive and quantitative detection of *S. pneumoniae* (2.0 CFU/mL for SERS, 3.6 × 10^2^ CFU/mL for photothermal mode) and *K. pneumoniae* (2.0 CFU/mL for SERS, 1.8 × 10^2^ CFU/mL for photothermal mode) in primary health‐care settings. We also verified the practical detection capability of the proposed LFA in clinical saliva samples, CuS‐Au based colorimetric enhanced and SERS LFA displayed 100% concordant with those of quantitative real‐time PCR (qPCR) for ten clinical saliva samples and five healthy control saliva samples. Notably, compared with qPCR required nucleic acid extraction and amplification steps (at least 2 h), our LFA can detect saliva samples within 15 min without any pretreatment. These results indicated that our CuS‐Au based LFA showed great potential for rapid, sensitive, and accurate tools for pathogens multi‐mode diagnosis in various POCT or resource‐limited settings.

As demonstrated by the aforementioned results, based on the plasmonic nanozyme, SERS/catalytic colorimetric/photothermal multimodal LFA can be fabricated for various applications. Compared with traditional single‐mode LFA, multimodal LFA can provide multiple signal outputs which allow for mutual calibration thus the detection accuracy can be improved. Moreover, the multimodal LFA integrates instrument‐free visual mode and SERS/temperature signals based quantitative modes, which can cover a variety of scenarios flexibly.

## 2 Results and Discussion

### 2.1 Schematic Illustration of Ligand‐Driven CuS‐Au Heterostructure Synthesis and Multimode LFA Based Bacterial Detection

**Scheme 1:**
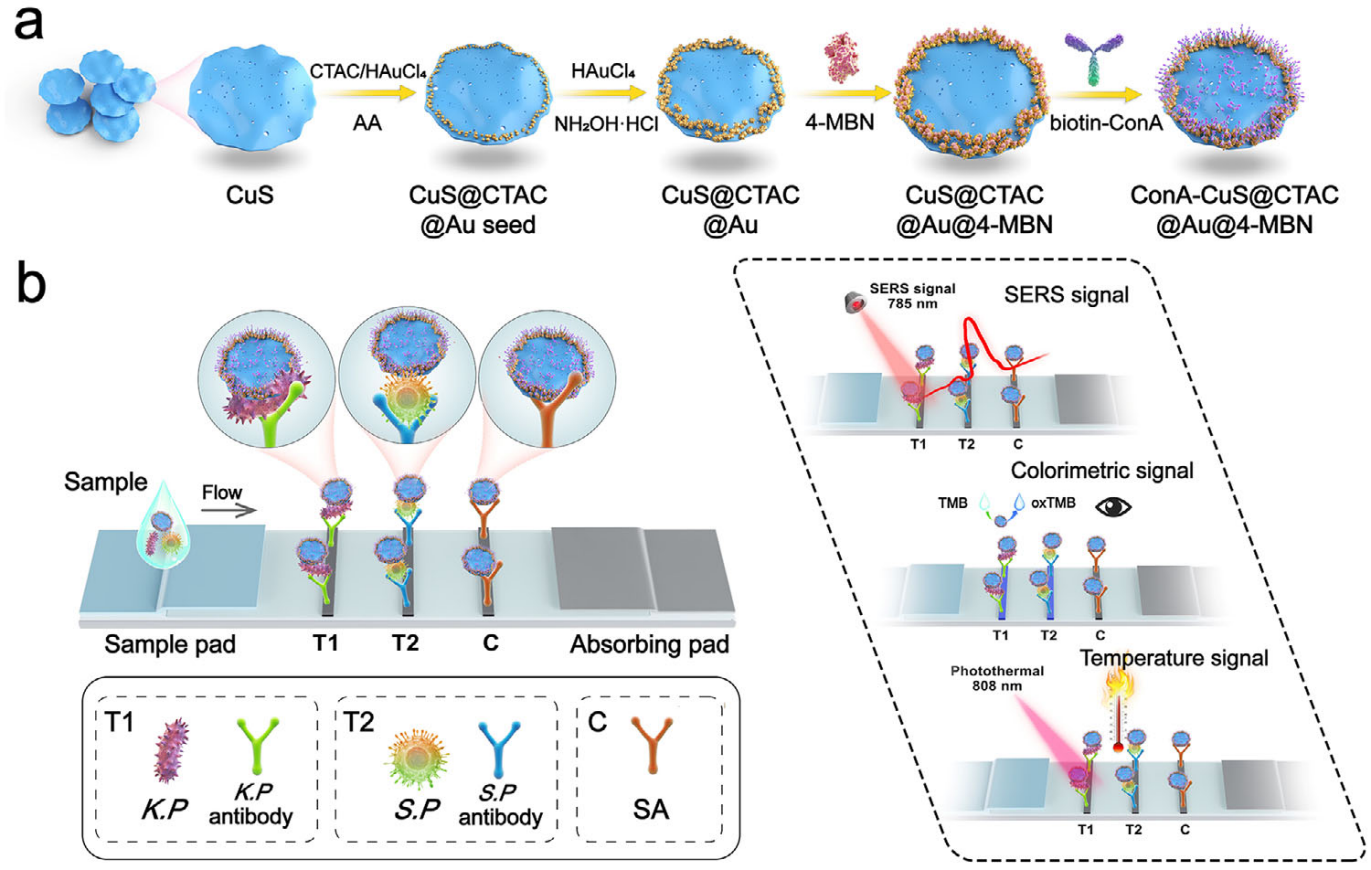
Principle diagrams of a) the synthesis procedure of CTAC regulated CuS‐Au heterostructure and the SERS reporter and biotin‐ConA modification as signal nanotags, b) the CuS‐Au nanotags assembled LFA for the SERS/colorimetric/photothermal multimode visual/quantitative detection of *S. pneumoniae* and *K. pneumoniae*.

The synthesis of ligand‐driven CuS‐Au heterostructure was illustrated in **Scheme** **1**a, firstly, CuS nanodiscs were prepared by a common one‐pot method, then ligands were modified on the surface of CuS, then Au^3+^ precursor and ascorbic acid (AA) was added to generate Au seeds through a site‐selective growth protocol regulated by CTAC, futher, Au^3+^ precursor and NH_2_OH·HCl were added to produce Au nanostarts in‐situ of the Au seeds. Finally, to obtained CuS‐Au heterostructures were modified by Raman reportor 4‐MBN and bacterial capture molecule biotin‐Concanavalin A (ConA) respectively. The principle of multimode LFA was illustrated in Scheme 1b. Here, broad‐spectrum capture of bacteria was performed by modifying biotinylated **Con A**. Firstly, biotin‐ConA modified CuS@CTAC@Au@4‐MBN nanotags can bind *K. pneumoniae* and *S. pneumoniae* and then the mixture was loaded onto the LFA, where the solution migrated along the strip due to capillary forces. The feasibility of the strips was verified by modifying *K. pneumoniae* antibodies on test line T1, *S. pneumoniae* antibodies on test line T2, and streptavidin (SA) on the control line C. The nanocomposite/bacterial complex can be specifically captured by *K. pneumoniae* and *S. pneumoniae* recognized antibodies on the T1 and T2 lines. The SERS/colorimetric/temperature signals can be readout on the T1 and T2 lines by visual qualitative detection and SERS or temperature quantitative determination.

### 2.2 Ligands‐Driven Site‐Selective Growth Protocol and Dual‐Activity of Cus‐Au Heterostructures

**Figure 1:**
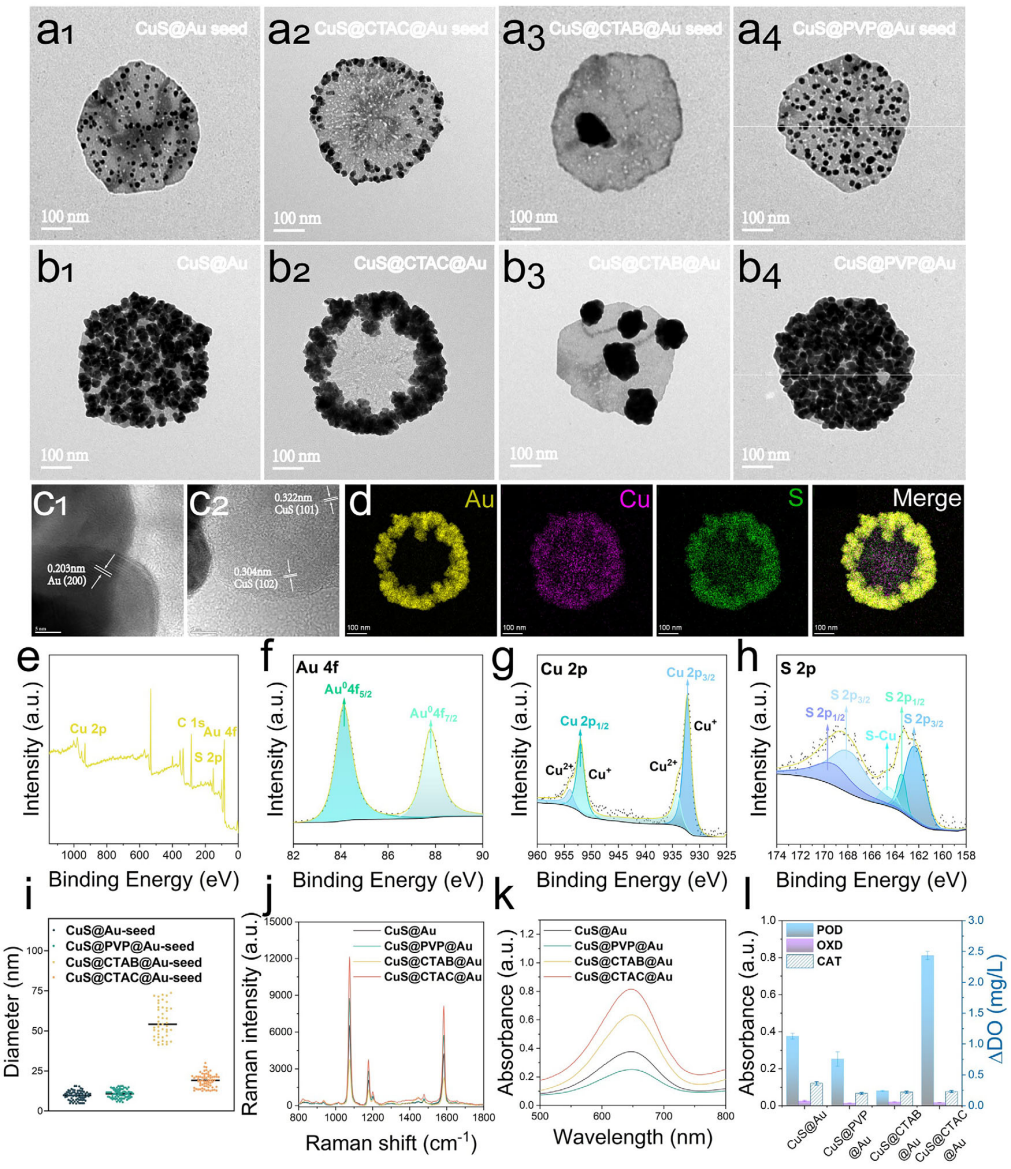
a) TEM images of CuS@Au seed regulated by different ligands. b) TEM images of the corresponding CuS‐Au nanoparticles in‐situ growth on Au seeds. c,d) Crystalline structure analysis and corresponding elemental mapping images of CTAC regulated CuS‐Au shown in inset (b2). e–h) XPS results of the CTAC regulated CuS‐Au. i) The size distribution of Au seed with different ligands (n = 50). j,k) SERS spectra and POD‐mimic activity (UV–vis absorption spectra with TMB‐H_2_O_2_) of CuS‐Au regulated by different ligands. l) POD/OXD/CAT‐mimic enzyme activity of CuS‐Au nanozymes regulated by different ligands, (mean ± sd., n = 3).

Firstly, we studied the morphological‐regulated effect of three commonly used ligands including N‐hexadecyltrimethylammonium chloride (CTAC), hexadecyltrimethylammonium bromide (CTAB), and polyvinyl pyrrolidone (PVP) (the corresponding structures were presented in Figure S1, Supporting Information) to the morphology of CuS‐Au heterostructures. The three ligands were modified on the surface of CuS nanodisc to regulate the Au seeds growing on the surface of CuS, afterward, Au seeds sequentially in situ growing to form bigger Au nanoparticles. The zeta potentials of CuS before and after modified with different ligands are illustrated in Figure S2 (Supporting Information), the obvious changed zeta potentials indicated the successful modification of different ligands. The transmission electron microscopy (TEM) images of CuS@ligands@Au seed and the corresponding CuS@ligands@Au nanoparticles were illustrated in **Figure** **1**a,b. It can be seen that without any ligands adding, Au seeds uniformly distributed on the surface of CuS nanodisc (the TEM images of CuS nanodisc are shown in Figure S3a, Supporting Information) and continued to form Au nanoaprticles arrays on the whole surface of CuS (Figure a1, b1), for CTAC, it indicated that CTAC can mediate the Au seeds edge‐epitaxially growing around the CuS nanodisc (Figure a2, b2), while for CTAB, Au seeds assembled to much bigger and infrequent nanoparticles and spread on the CuS nanodisc surface (Figure a3, b3). For PVP, Au seeds and Au nanoparticles can distribute more uniformly and compactly on the whole surface of CuS nanodisc compared with that without any ligands added (Figure a4, b4). All of the results indicated that by using of different ligands, Au nanoparticles can be mediately grown to form different CuS‐Au heterostructures. For PVP, which is usually used as stabilizer and dispersant in the growth of nanoparticles, more uniform Au nanoparticles distribution can be obtained.^[^ ^41^ ^]^ For CTAB and CTAC, both of them can interact with the electronegative CuS surface through trimethylammonium cation head groups (N^+^), however, CTAB(Br⁻) has bigger polarization ability and ionic radius than CTAC(Cl⁻), so that CTAB possessed higher affinity to CuS surface to form a dense adsorption layer on the whole surface of CuS, which can block the Au uniformly growing on the whole CuS nanodisc, while CTAC possessed weaker affinity to CuS and preferential absorbed on the main surface with low surface energy of CuS nanodisc, so that Au mainly grew around the annular edge of CuS nanodisc to form annular‐epitaxial CuS‐Au heterostructures.^[^ ^42^, ^43^, ^44^ ^]^ To validate the role of ligands in mediating the annular‐epitaxial growth of CuS‐Au heterostructures, we further investigated ligands CTAC and its homolog cetylpyridinium chloride (CPC), CTAB and its homolog cetylpyridinium bromide (CPB). As shown in Figure S4, Supporting Information, CPC (with Cl^⁻^) also displayed the ability to drive the annular‐epitaxial growth of CuS‐Au heterostructure, while by using CPB (Br^⁻^), similar to CTAB, only few irregular big Au nanoparticles growth on the surface of CuS. This result demonstrates that ligands with similar long‐chain alkyl and Cl^−^ properties (CTAC and CPC) may regulate the spatially selective annular‐epitaxial growth of Au on CuS.

Previous studies have shown that light conditions can affect the binding energy of the material.^[^ ^45^ ^]^ To exclude photo‐driven effects, Au reduction on CuS@CTAC substrates was performed under complete darkness and ambient illumination. TEM imaging (Figure S5, Supporting Information) revealed identical edge‐specific Au growth patterns under both conditions, confirming that nucleation is governed by CTAC's molecular capping effect rather than light‐dependent processes.

We also confirmed the crystalline structure of CuS@CTAC@Au (Figure b2) by HRTEM images (Figure 1c1,c2) and selected area electron diffraction (SAED) pattern (Figure S6, Supporting Information), obvious Au (200) and CuS (102101) characteristic lattice planes can be found. The energy‐dispersive X‐ray (EDX) element mapping (Figure 1d) also confirmed the annular epitaxial growth of Au on the CuS nanodisc. Moreover, the UV−vis absorption spectra of CuS@Au showed a red‐shift than that of CuS (Figure S7, Supporting Information), indicating the successful synthesis of CuS@Au. X‐ray photoelectron spectroscopy (XPS) spectrum was also used to investigate the composition and structure of CuS@CTAC@Au, as shown in Figure 1e, elements of Au, Cu, and S can be detected clearly, moreover, the high‐resolution XPS spectra of Au 4f, Cu 2p and S 2p was also analyzed. As illustrated in Figure 1f, two photoelectron peaks at 87.8 and 84.1 eV correspond the Au 4f_7/2_ and 4f_5/2_ can be found,^[^ ^46^ ^]^ the 952 and 932.2 eV confirm the presence of Cu 2p_1/2_ and 2p_3/2_ in Figure 1g, moreover, the ratio of Cu^+^ was much higher than that of Cu^2+^, which indicated the high POD‐mimic activity,^[^ ^47^ ^]^ in the Figure 1h, two photoelectron peaks at 168.6 eV and 163.2 eV corresponding to S 2p_1/2_ and S 2p_3/2_ respectively can be found, moreover, the peak at 164.6 eV which indicated the S‐Cu bond region can be seen clearly.^[^ ^48^ ^]^ Meanwhile, we conducted powder X‐ray diffraction (XRD) and Raman spectroscopy characterizations on the CuS nanodiscs and CuS‐Au heterostructures. The XRD analysis in Figure S8a (Supporting Information) showed that distinct Au characteristic diffraction peaks (JCPDS No. 04–0784) 38.2° (111), 44.5° (220), 64.6° (200), 77.5° (311) and CuS diffraction peaks (JCPDS No. 06–0464) 27.6° (101), 31.7° (103), 32.8° (006), 52.7° (108), 58.6° (203), 59.3° (116), can be seen clearly, indicating the successful synthesis of Au‐CuS composite. The Raman spectroscopy of CuS and CuS@CTAC@Au heterostructures were also detected, as shown in Figure S8b (Supporting Information), the CuS exhibits a strong characteristic peak at 475 cm⁻¹, attributed to the symmetric stretching vibration of S‐S bond.^[^ ^49^ ^]^ However, in the heterostructure, the characteristic peak at 475 cm^−^¹almost disappeared, two new characteristic peaks at 1385 cm^−^¹ (C‐N stretching vibration, CTAC) and 1570 cm^−^¹ (C = O stretching vibration, PVP) can be seen clearly.^[^ ^50^, ^51^ ^]^ That can be attributed to the SERS effect of plasmonnic Au to the ligand molecule such as CTAC and PVP used in the synthesis of CuS@CTAC@Au, the strong SERS peaks can mask the relative weak S‐S bond.

The size distribution of Au seeds regulated by different ligands was also shown in Figure 1i according to TEM images of Figure 1a, it can be seen that both of PVP and CTAC can mediate uniform Au seeds, while for CTAB, inhomogeneous Au seeds with bigger sizes can be found, which was consistent with the finally CuS‐Au heterostructures as shown in Figure 1b.

Next, in order to verify whether CuS‐Au heterostructures can be used as SERS and nanozyme tags, the SERS spectra (using 4‐MBN as a Raman reporter, using 1074cm^−1^ as characteristic peak) and the POD‐mimic activity (using TMB+H_2_O_2_ chromogenic system) of different ligands regulated heterostructures named CuS@X@Au (X means CTAB, CTAC, and PVP) were detected, CuS@Au without any ligands was used as a control. Firstly, we have evaluated the POD activity and SERS properties of CuS nanosheets. As shown in Figure S3 (Supporting Information), CuS nanosheets display obvious POD activity but no SERS property. As shown in Figure 1j,k, CuS@CTAC@Au displayed superior SERS intensity and catalytic activity than the others. The reason can be attributed to the structure of CuS‐Au heterostructures. For annular‐epitaxial CuS@CTAC@Au, bare CuS in the center can provide superior POD‐mimic activity, while Au nanoflowers closely arrayed around the CuS, abundant surface plasmon resonance (SPR) hot‐spots can be obtained to improve the Raman intensity of 4‐MBN modified on the Au nanostars.^[^ ^52^ ^]^ While for CuS@CTAB@Au, large area of bare CuS can provide obvious POD signals, however, the sparsely distributed Au nanoparticles cannot provide enough hotspots to induce obvious SERS signals, for CuS@PVP@Au and CuS@Au, only obvious SERS signals can be found and almost no POD activity can be detected because that most of POD‐mimic CuS surface was covered by Au nanoparticles. In a word, we found that only by using of CTAC, the annular‐epitaxial growth of CuS‐Au heterostructures can be obtained to produce both of superior POD‐catalytic and SERS signals, which can be promising dual‐mode tags in biosensing.

Further, the multi‐enzyme activities (POD/OXD/CAT) of these nanocomposites were also studied, as shown in Figure 1l, CuS@CTAC@Au displays superior POD‐mimic activity but almost no OXD and CAT‐mimic activity, it is beneficial for the accurate detection of targets because in the usual used POD‐mimic assay, the OXD‐mimic activity of the nanozyme tags can invert uncertain dissolved O_2_ to O^2·‐^ which directly converts colorless TMB to blue oxTMB, the CAT‐mimic activity of the nanozyme tags can consume H_2_O_2_ to produce O_2_ directly, the unspecific multi‐enzyme activities can induced interference‐background signals which can disturb the detection sensitivity and repeatability.^[^ ^53^, ^54^ ^]^ Finally, the SERS signal was used to monitor the stability of the CuS@CTAC@Au nanozyme due to the precise quantitative characteristic of SERS intensity. As shown in Figure S9a,b (Supporting Information), the SERS signals of nanozyme were detected after storage at 1, 10, 20, and 30 days respectively, the SERS signals changed little with a relative standard deviation (RSD) value 5.1%, which indicated that the CuS@CTAC@Au can be stable after long time storage. Further, to verify the reproducibility of CuS@CTAC@Au from batch to batch, three independent batches of samples were prepared under the same scheme, and the RSD value of the three SERS signals (Figure S9c,d, Supporting Information) was 4.5%, indicating high consistency between batches. Therefore, the CuS@CTAC@Au possessed superior stability and reproducibility.

### 2.3 The CTAC Regulated Site‐Selective Growth Protocol and the Corresponding Catalysis/SERS Performance

**Figure 2:**
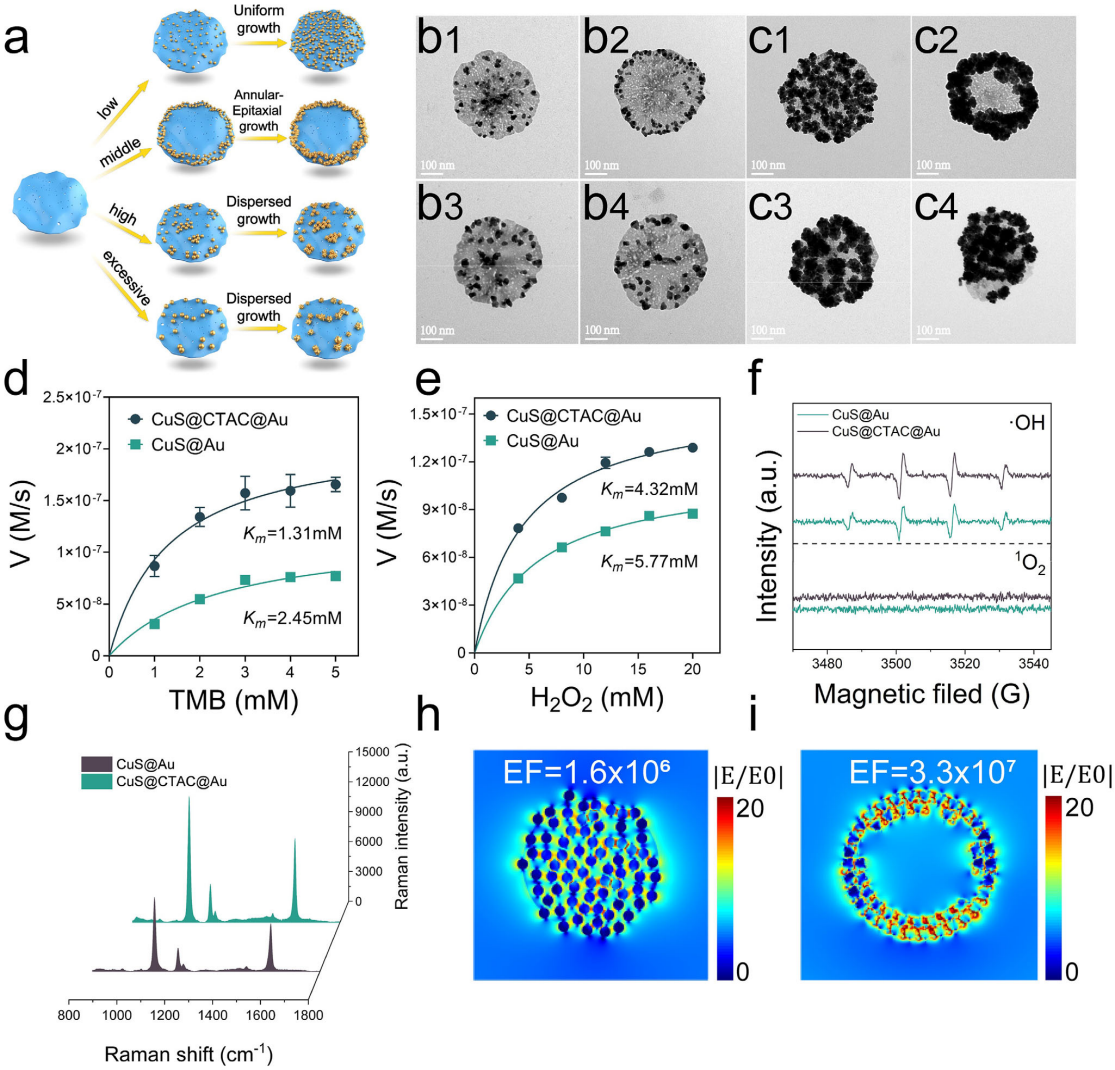
a) Four growth behaviors of Au on CuS regulated by different CTAC concentrations. b,c) TEM images of four growth behaviors of Au seeds and the corresponding Au nanoparticles under the CTAC regulations. d,e) Steady‐state kinetic analysis of CuS@CTAC@Au and CuS@Au with TMB and H_2_O_2_ respectively (mean ± sd., n = 3). f) ESR spectra of CuS@CTAC@Au and CuS@Au with or without H_2_O_2_ to explain the •OH and ^1^O_2_. g) SERS spectra of 4‐MBN modified CuS@CTAC@Au and CuS@Au. h,i) FDTD simulations of CuS@Au and CuS@CTAC@Au electromagnetic fields.

The detailed effect of CTAC on the site‐selective growth of Au on the CuS nanodisc was first investigated and the schematic illustration was firstly shown in **Figure** **2**a. It can be seen that with low concentration of CTAC (100 µM), the packing density of CTAC molecules on the CuS nanodisc was low and made negligible effect on the growth of Au seeds and the subsequent Au nanoparticles (Figure 2b1–c1), when using of appropriate concentration (1 mm), CTAC mainly packed on the center surface of CuS, the steric hindrance can inhibit Au seeds growing on the central surface, thus annular‐epitaxial CuS‐Au heterostructures can be obtained (Figure 2b2–c2). With continually increased CTAC (5 mm), more CTAC packing both on the center and edge of CuS, the larger steric hindrance restricted the Au seeds uniformly growing and led to much bigger but heterogeneous Au nanoparticles distribution on the surface of CuS (Figure 2b3–c3), when CTAC increasing to 10 mm, the heterogeneity of Au nanoparticles turned to more prominent (Figure 2b4–c4). The size distribution of Au seeds at different CTAC concentrations was also confirmed and shown in Figure S10 (Supporting Information), it can be seen that the size of Au seeds gradually increased with the increasing concentration of CTAC, this result confirmed that CTAC can regulated the site‐selective growth and size distribution of Au seeds, which continued effect the growing of Au nanoparticles.

We characterized the modification amount of CTAC on CuS nanodiscs under different CTAC modification by using of Cl⁻‐quantify ion chromatography. Specifically, CuS nanodiscs modified with different concentrations of CTAC (0.1, 1, 5, 10 mm) were subjected to ultrasonication to detach CTAC from the CuS surface. Then, the solution was ultrafiltrated to obtain CTAC filtrate, the filtrate was then detected by Cl⁻‐quantify ion chromatography and the Cl⁻ concentration was used to represent the amount of CTAC modification on CuS. As shown in Figure S11 (Supporting Information), the Cl⁻ concentration gradually increased with increasing CTAC modification concentrations from 0.1 to 5 mm, and almost reached a plateau at 5 mm. This result indicates that CTAC ligands can be near‐saturated at the modification concentration of 5 mm on the surface of CuS. Further studies on the tolerance of CuS nanodiscs to CTAC concentrations were performed by using high CTAC concentrations (25, 50, and 100 mm) to modify CuS respectively. As shown in Figure S12 (Supporting Information), after incubation with CTAC at all concentrations, the size, edge morphology, and layered structure of CuS nanosheets remain consistent, confirming the superior compatibility between CTAC ligands and CuS nanodiscs (Figure S12, Supporting Information).

The effect of HAuCl_4_ concentration was also investigated. As shown in Figure S13a (Supporting Information), at appropriate CTAC concentration (1 mm), Au nanoparticles grew from edge to center on the surface of CuS. The corresponding SERS and POD‐mimic activity of the CuS@CTAC@Au with different concentrations of HAuCl_4_ were also studied, as shown in Figure S13b‐7c (Supporting Information), the optimal POD catalytic activity and SERS intensity of Cus‐Au can be obtained when the concentration of HAuCl_4_ was 200 µm for Au nanoparticles growing on the CuS, at lower or higher HAuCl_4_ concentrations, much weaker SERS signals or much weaker POD catalytic activity can be induced, which was unusable as dual‐mode signal tags.

Further, the POD‐mimic catalytic activity and SERS performance of the optimal CuS@CTAC@Au was studied. As shown in Figure 2d,e, steady‐state kinetics detection was performed at different concentrations of TMB and H_2_O_2_, according to the Michaelis‐Menten model, the *K_m_* of CuS@CTAC@Au to TMB and H_2_O_2_ was 1.3 and 4.3 mm respectively, which was lower than that of CuS@Au, indicating that CuS@CTAC@Au has better affinity to TMB and H_2_O_2_ than CuS@Au. In addition, we compared the CuS@CTAC@Au nanozyme developed in this study with newly reported Cu‐Au based POD‐mimic nanozymes. As shown in Table S1 (Supporting Information), CuS@CTAC@Au exhibits superior *K_m_* and *V_max_* values and outstanding POD‐mimic activity. The electron paramagnetic resonance (EPR) spectroscopy was employed to investigate the generation of · OH (with H_2_O_2_) and ^1^O_2_ (without H_2_O_2_) by using of 5, 5‐dimethyl‐1‐pyrroline n‐oxide (DMPO) as a capturer at pH 4 (Figure 2f), it demonstrated CuS@CTAC@Au can induced more · OH than CuS@Au, however, no ^1^O_2_ can be found in the presence of CuS@CTAC@Au, indicating that CuS@CTAC@Au indicated no OXD‐mimic activity and can be used as specific POD‐mimic nanozyme for background interference‐free biosensing. We further verified the POD‐mimic catalytic activity of CuS@CTAC@Au nanozymes with pH from 1 to 12, as shown in Figure S14 (Supporting Information), CuS@CTAC@Au display excellent catalytic activity at pH 2–4. The SERS performance of CuS@CTAC@Au was also verified. As shown in Figure 2g, the SERS intensity of 4‐MBN modified CuS@CTAC@Au increased obviously than that of CuS@Au, the finite‐difference time‐domain (FDTD) simulation was conducted to investigate the corresponding near‐field electromagnetic (EM) field distributions. As shown in Figure 2h,i, intense localized surface plasmon resonance hot‐spots can be found in the compact junctions between Au nanoparticles around the edge of CuS for the CuS@CTAC@Au structure, while for CuS@Au, interval arrangement of Au can induce weaker localized surface plasmon resonance hot‐spots, the EM field enhancement factors (EF) of CuS@Au and CuS@CTAC@Au was calculated as 3.3 × 10^7^ and 1.6 × 10^6^ respectively,^[^ ^55^ ^]^ which confirmed the higher SERS enhancement signals of CuS@CTAC@Au, the EF of CuS was only 10.5 (Figure S15, Supporting Information), it indicated that CuS nanodisc has almost no SERS enhancement ability.

### 2.4 Evaluation of Photothermal and Plasmon‐Enhanced Nanozyme Catalytic Activity

**Figure 3:**
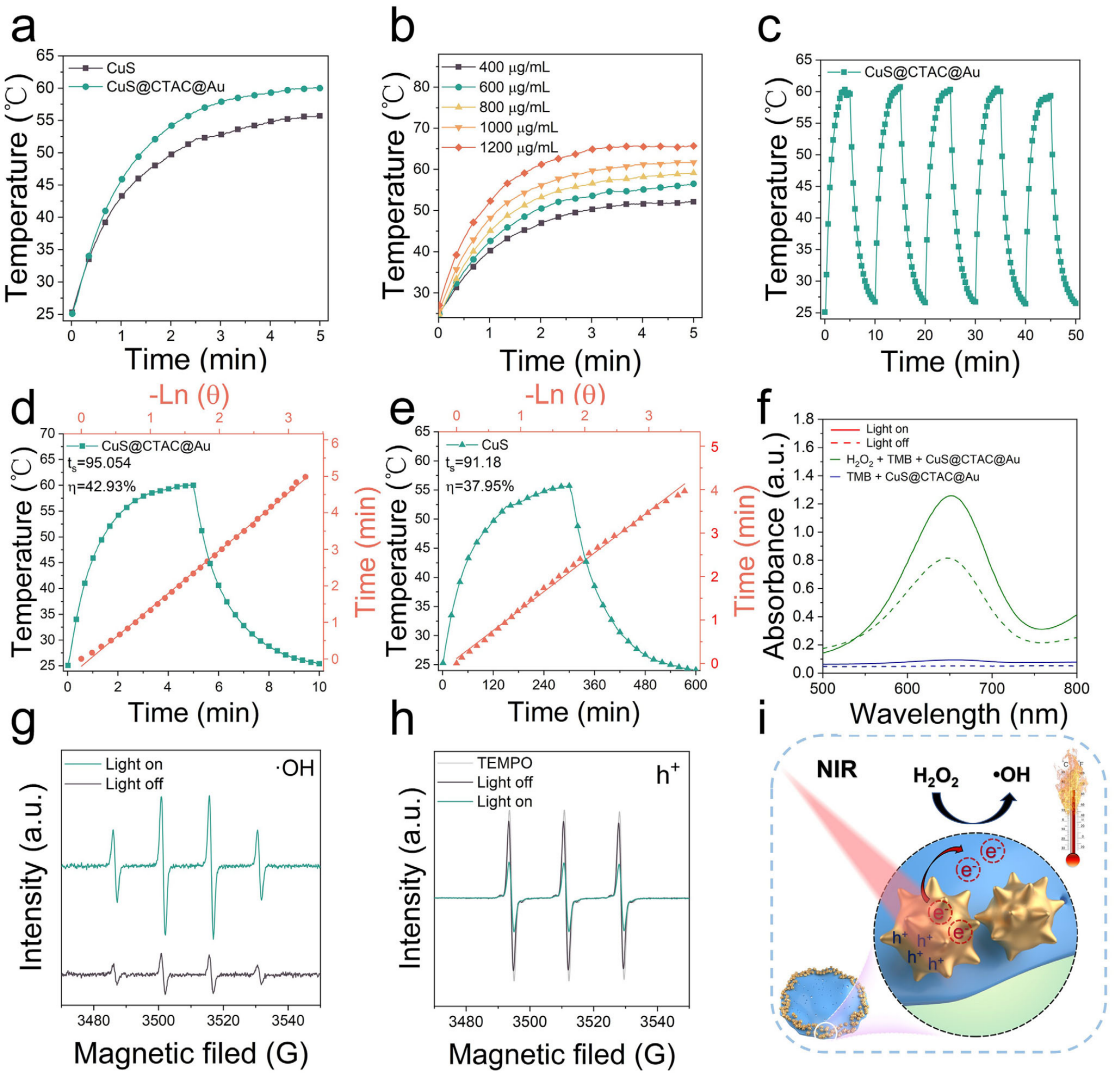
a) Temperature curves of CuS and CuS@CTAC@Au under the 808 nm laser irradiation. b) Temperature curves of CuS@CTAC@Au with different concentrations under 808 nm laser irradiation. c) Photothermal stability of CuS@CTAC@Au for five heating‐cooling cycles. d,e) Temperature profile of CuS@CTAC@Au and CuS under photothermal heating and natural cooling and the corresponding photothermal conversion efficiency (η). f) The UV–vis absorption spectra of CuS@CTAC@Au in the H_2_O_2_ and TMB chromogenic agents with or without 808 nm laser irradiation. g,h) ESR spectra of DMPO/•OH spin adduct and TEMPO/h^+^ generated by CuS@CTAC@Au and H_2_O_2_ with or without 808 nm laser irradiation. i) The schematic of photothermal property and plasmon‐enhanced POD‐mimic enzyme activity of CuS@CTAC@Au.

The photothermal property of CuS@CTAC@Au was also studied and shown in **Figure** **3**a, after 5 min irradiation by 808 nm laser (1.5 W/cm^−2^), the temperature of CuS@CTAC@Au solution was 4.3 °C higher than that of CuS, indicating that CuS@CTAC@Au possesses better photothermal property than that of CuS (Figure S16, Supporting Information), which is consistent with previous result.^[^ ^38^ ^]^ The photothermal curves shown in Figure 3b and Figure S17 (Supporting Information) indicated that the photothermal property of CuS@CTAC@Au was concentration and laser power density dependent. After 5 cycles of heating and cooling, the CuS@CTAC@Au displays photothermal stability (Figure 3c), as shown in Figure 3d,e, the photothermal conversion efficiency(η) of CuS@CTAC@Au is 42.9%, which is better than that of CuS (37.9%), therefore, CuS@CTAC@Au possesses good photothermal property and can be a potential tag for photothermal signals detection.

The plasmon‐enhanced catalytic activity of CuS@CTAC@Au was also studied. As shown in Figure 3f, after 5 min irradiation by 808 nm laser (1.5 W/cm^−2^), the POD‐mimic activity (evaluated by UV‐visible absorption peak of oxTMB at 652 nm in the presence of H_2_O_2_) increased obviously, while for the OXD‐mimic activity (evaluated by UV‐vis absorption peak of oxTMB at 652 nm in the absent of H_2_O_2_), almost no signals can be found, this result indicates that CuS@CTAC@Au really displays plasmon‐enhanced POD‐catalytic activity and almost no OXD‐catalytic activity under irradiation, it can be advantage for accurate colorimetric detection because the background signals induced by uncertain dissolved oxygen in sample solutions (OXD can invert O_2_ to O_2_ **^·‐^** which directly converts colorless TMB to blue oxTMB) can be eliminated. The electron spin resonance (ESR) spectra of CuS@CTAC@Au and H_2_O_2_ was also detected (Figure 3g), it can be seen that under 808 nm laser, more ·OH can be detected, which confirm the enhanced POD‐mimic activity by plasmonic irradiation. In addition, the spin label 2, 2, 6, 6‐tetramethylpiperidin‐1‐oxy (TEMPO) was used to characterize the presence of hot charge carriers. It is known that TEMPO can generate an ESR signal and the signal can be quenched after TEMPO capturing hot electrons. As shown in Figure 3h, the characteristic peak intensity of TEMPO in the CuS@CTAC@Au and H_2_O_2_ solution decreased slightly than that in the H_2_O_2_ solution, moreover, the characteristic peak intensity of TEMPO decreased obviously after 808 nm illumination. This result indicated that plasmonic irradiation can really improve the hot electron generation in the CuS@CTAC@Au and H_2_O_2_ catalytic system.^[^ ^56^ ^]^ The photothermal property and plasmon‐enhanced POD‐catalytic mechanism of CuS@CTAC@Au can be illustrated in Figure 3i, upon the NIR excitation, increasing e‐h^+^ pairs generated on the surface of CuS@CTAC@Au, the hot electrons can transfer from Au to CuS and accelerated the H_2_O_2_ decomposition to produce more ·OH, moreover, the temperature of CuS@CTAC@Au also increased under the plasmon excitation, thus the enhanced POD‐mimic activity can be attributed to the combined effect of plasmon‐induced e‐h^+^ pairs and the near‐field heating to boost the catalytic performance.

### 2.5 Feasibility of ConA Modified Cus@CTAC@Au Heterostructures Based LFA for *K. Pneumoniae* and *S. Pneumoniae* Recognition

**Figure 4:**
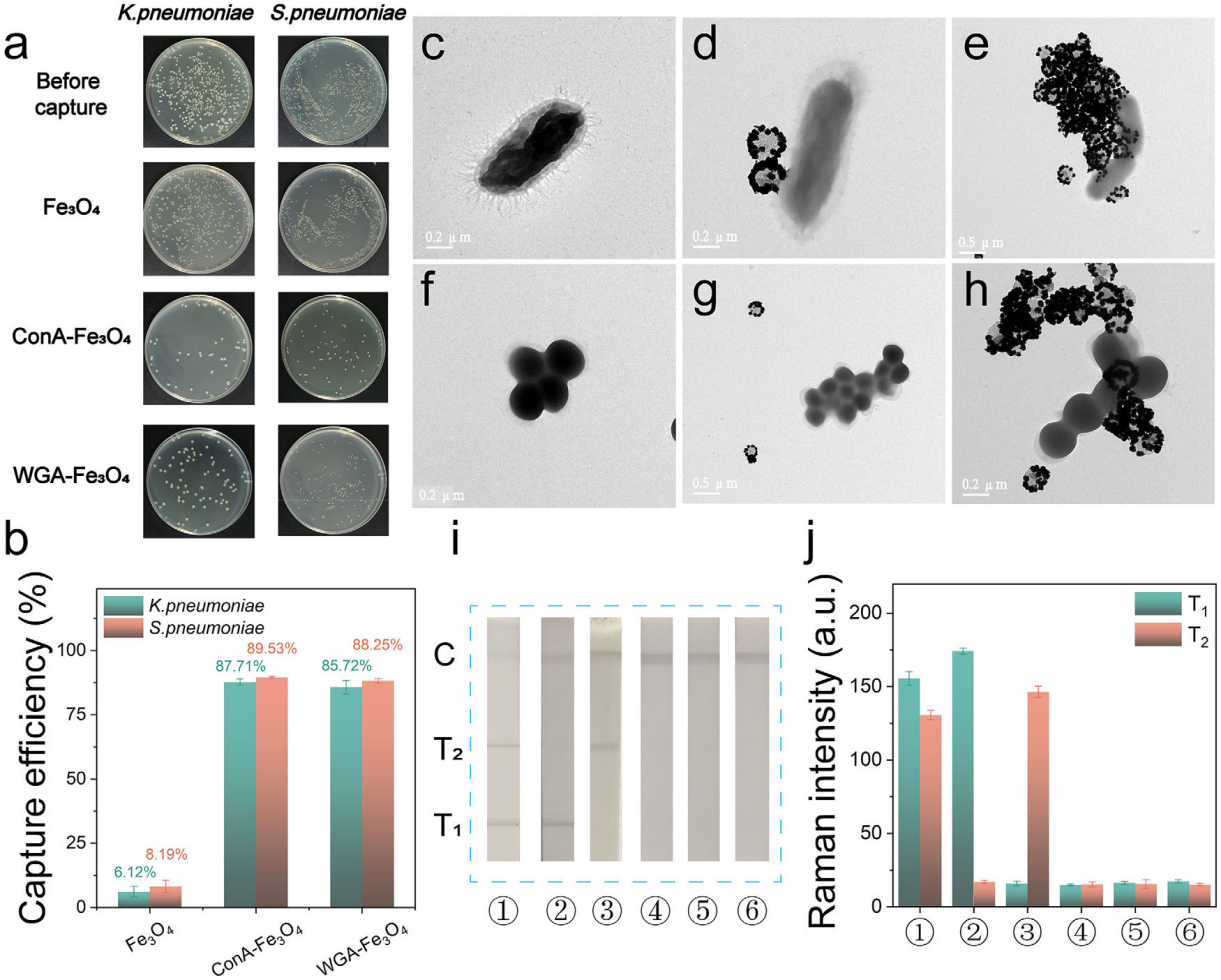
a) The photographs of the original colonies on LB‐agar plates of *K. pneumoniae* and *S. pneumoniae* and the amounts of remaining bacteria in the supernatant after captured by Fe_3_O_4_, Con A modified Fe_3_O_4_ and WGA modified Fe_3_O_4_, b) the corresponding capture efficiency according to colonies of (a). c–e) TEM images of *K. pneumoniae* (c), *K. pneumoniae* binding with CuS@CTAC@Au (d), and with ConA‐CuS@CTAC@Au (e). f–h) TEM images of *S. pneumoniae* (f), *S. pneumoniae* binding with CuS@CTAC@Au (g), and with ConA‐CuS@CTAC@Au (h). i,j) LFA photos and the corresponding SERS intensity of the T1 and T2 lines in the presence of ① *K. pneumoniae* and *S. pneumoni*,② *K. pneumoniae*, ③ *S. pneumoniae*, ④ *E. coli*, ⑤ *S. aureus*, ⑥ PBS (for all of the bacteria, the same concentration of 10^5^ cells/mL was used).

It has been reported that phytolectin such as ConA^[^ ^57^ ^]^ and wheat germ agglutinin (WGA)^[^ ^58^ ^]^ can be universal capture tools for various pathogen bacteria, however, few researches have reported whether the lectins can be used for *K. pneumoniae* and *S. pneumoniae* capture. Here, we first verified the affinity of two most commonly used lectins Con A and WGA to *K. pneumoniae* and *S. pneumoniae*, by using Con A or WGA modified Fe_3_O_4_ nanoparticles. First, *K. pneumoniae* and *S. pneumoniae* solutions (10^5^ CFU/mL for each) were incubated with ConA or WGA modified Fe_3_O_4_ respectively, after magnetic separation, the plate culture method was used to quantify the residual bacteria in the supernatant, as shown in **Figure** **4**a,b, the capture efficiency ((the difference value of original amount and the remained amount of bacteria)/ the original amount of bacteria) of Con A modified Fe_3_O_4_ was 89.5% for *S. Pneumoniae* and 87.7% for *K. Pneumoniae*, the capture efficiency of WGA modified Fe_3_O_4_ was 88.2% for *S. Pneumoniae* and 85.7% for *K. Pneumoniae*, it can be seen that both Con A and WGA possess good affinity to *S. Pneumoniae* and *K. Pneumoniae*, we chose ConA to modified on the CuS@CTAC@Au as the specific recognition molecule in the subsequent experiments. As shown in the TEM images of Figure 4c–h, ConA‐CuS@CTAC@Au could bind onto the surface of *K. Pneumoniae* and *S. Pneumoniae* clearly (Figure 4e,h), while for CuS@CTAC@Au only, almost no adherence of CuS@CTAC@Au with *K. Pneumoniae* and *S. Pneumoniae* can be found (Figure 4d,g). This result verified that ConA‐CuS@CTAC@Au can be used as high‐affinity SERS tags to label *S. Pneumoniae* and *K. Pneumoniae*. Subsequently, the feasibility of ConA‐CuS@CTAC@Au@4‐MBN based dual‐target LFA for *S. Pneumoniae* and *K. Pneumoniae* detection was verified. The photographs of the strips and the corresponding SERS signals on the T1 and T2 lines for different bacterial samples (sample 1: 10^5^ CFU/mL of *S. Pneumoniae*/*K. Pneumoniae*; Sample 2: 10^5^ CFU/mL *K. Pneumoniae*; Sample 3: 10^5^ CFU/mL *S. Pneumoniae*; Sample 4: 10^5^ CFU/mL *Staphylococcus aureus*; Sample 5: 10^5^ CFU/mL *E. coli*; Sample 6: PBS)were illustrated in Figure 4i,j, it can be seen that the LFA can simultaneously recognize *S. Pneumoniae* and *K. Pneumoniae* with high specificity (strip 1 to 3), while for other bacterial samples such as *S. aureus* and *E. coli*, no obvious black band and SERS intensity on the T lines can be detected(strips 4,5), here, PBS buffer was used as a blank control (strip 6). Then, several important factors such as the amount of SERS tags, the incubation time of tags and pathogens, the types of NC membranes, and the running buffers were optimized, SNR value (signal‐to‐noise ratio, PBS was used as blank control, *K. Pneumoniae* and *S. Pneumoniae* (10^5^ CFU/mL) were used as targets. The result was illustrated in Figure S18 (Supporting Information), it displayed that an optimized SNR value can be obtained when 2 µL of SERS tags (0.3 mg mL^−1^), 10 min incubation time of pathogens and tags, CN95 membrane and PBST (1% Tween‐20+10 mm Ca^2+^/Mg^2+^ +1.5% not fat dry milk) buffer are used respectively. We also optimized the wavelength of the Raman excitation laser (532, 633, and 785 nm). As shown in Figure S19 (Supporting Information), by using of 785 nm excitation laser, superior SERS signals can be found clearly, moreover, 785 nm excitation laser can reduce the fluorescence background signals especially in biological samples, thus 785 nm excitation laser was used in this research.

### 2.6 Performance Evaluation of the Multimode LFA for Pathogens Detection

**Figure 5:**
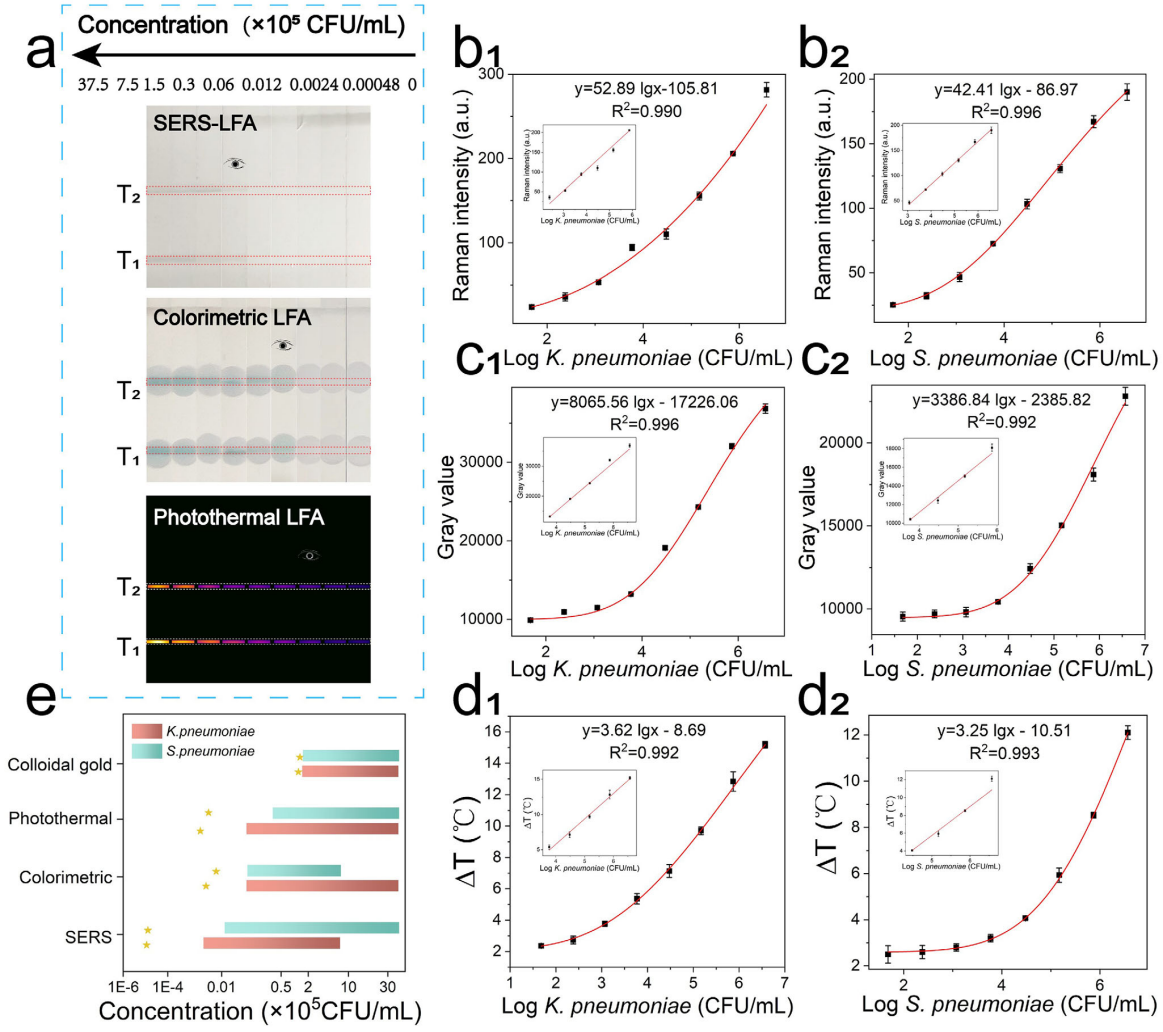
a) Photographs of CuS@CTAC@Au driven LFA for SERS, catalytic colorimetric and photothermal detection of different concentrations of *K. pneumoniae* and *S. pneumoniae*. b–d) Regression analysis and linear relationship of the SERS intensity (b), colorimetric intensity (c), and temperature (d) of the T1(*K. pneumoniae*) and T2 (*S. pneumoniae*) corresponding to LFAs shown in inset (a). e) Cooperation of the detection sensitivity and linear range among SERS/colorimetric/temperature three modes, common colloidal gold LFA was used as a control, the star marks represent the LOD value and the horizontal bars represent the detection range. Data are presented as mean ± s.d. (n = 3).

The analytical performance of the ConA‐CuS@CTAC@Au@4‐MBN based multimodal LFA was evaluated. First, target pathogens (T1: *K. pneumoniae*; T2: *S. pneumoniae*) with different concentrations from 0 to 3.75 × 10^6^ CFU/mL were incubated with ConA‐CuS@CTAC@Au@4‐MBN for 10 min, and then the mixture was loaded onto the strip for detection. **Figure** **5**a shows the photographs of LFA for SERS, catalytic colorimetric, and photothermal detection of different concentrations of *K. pneumoniae* and *S. pneumoniae*. It indicated that before colorimetric enhancement, ≈3.0 × 10^4^ CFU/mL can be visually observed, after colorimetric catalysis (by TMB+H_2_O_2_ system), visual concentration decreased to ≈1.2 × 10^3^ CFU/mL, about 25‐fold sensitivity improvement can be obtained. For photothermal images analysis, visual sensitivity was ≈2.4 × 10^2^ CFU/mL. Quantitative analysis was also performed based on the multimode signals of SERS, colorimetric, and temperature. As shown in Figure 5b–d, the detection limit (LOD) of SERS/colorimetric/temperature was 2.0/2.9 × 10^2^/1.8 × 10^2^ CFU/mL for *K. pneumoniae* (T1 line) and 2.0/6.5 × 10^2^/3.6 × 10^2^ CFU/mL for *S. pneumoniae* (T2 line) respectively, while for conventional colloidal gold test strips, only 1.2 × 10^5^ CFU/mL of *K. pneumoniae* and 1.3 × 10^5^ CFU/mL of *S. pneumoniae* can be detected (Figure S20, Supporting Information). The LOD values and detection ranges of the three modes are listed in Figure 5e, it can be seen that compared with common Au LFA, all of the three modes display better sensitivity and expanded detection range, we also compared our multimode LFA with other LFA based *K. pneumoniae* or *S. pneumoniae* biosensors, as shown in Table S2 (Supporting Information), many advantages such as high sensitivity, wide detection range, three‐way signal output capability and optional signal output mode can be demonstrated in our assay. More importantly, this is the first rapid LFA method for *K. pneumoniae* and *S. pneumoniae* synchronous recognition.

### 2.7 Application of the Constructed LFA in Clinical Samples

**Figure 6:**
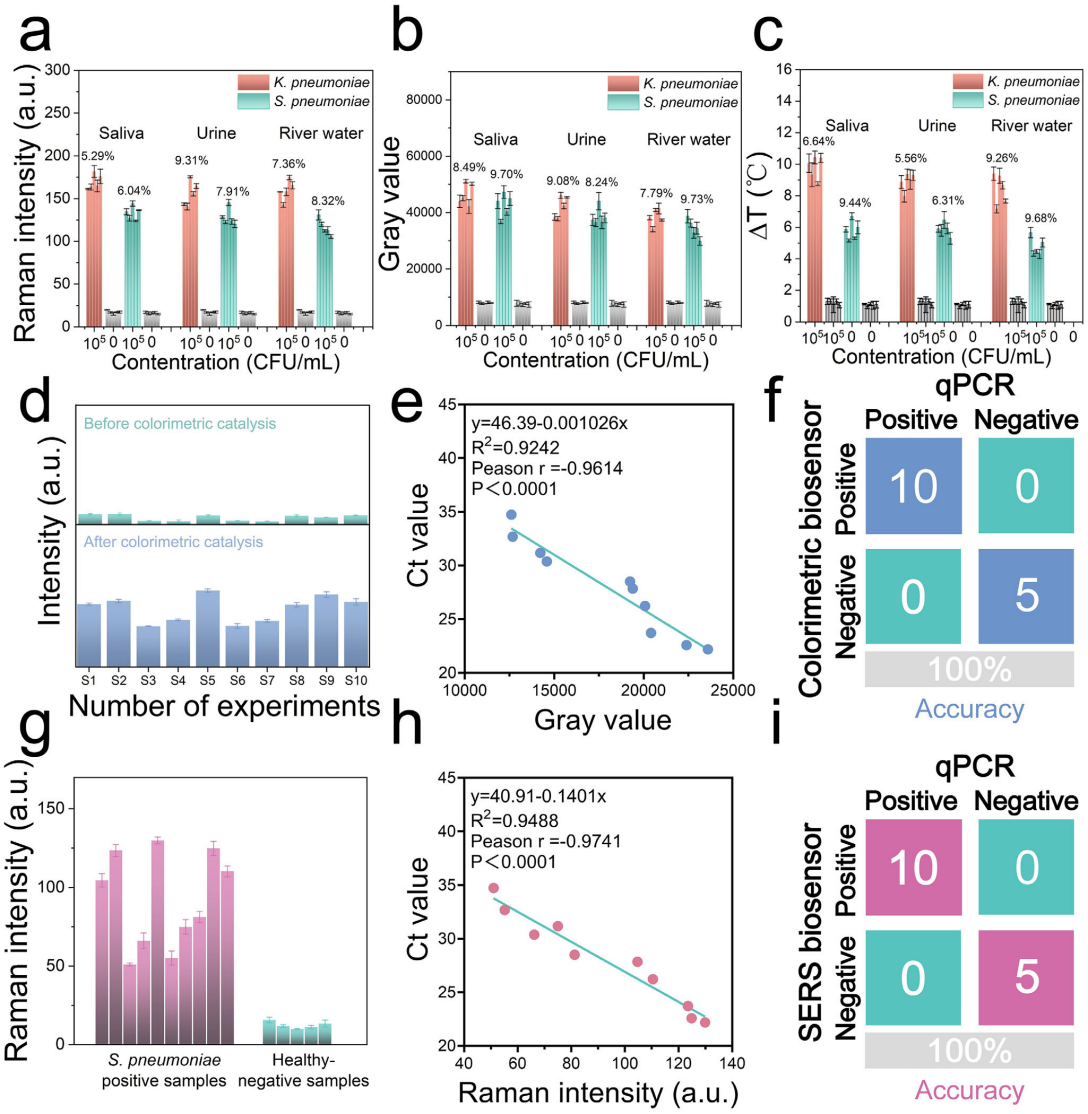
a–c) Reliability analysis of the SERS/colorimetric/photothermal three‐mode LFA for *K. pneumoniae* and *S. pneumoniae* (10^5^ CFU/mL for each) spiked in saliva, urine, and river water respectively. d,e) Colorimetric intensity and the consistency of colorimetric intensity and standard qPCR results for ten positive clinic samples. f) Evaluation of the sensitivity and specificity of nanozyme colorimetric‐LFA compared with standard qPCR using a confusion matrix. g,h) SERS intensity and the consistency of SERS intensity and the standard qPCR results for 10 positive clinic samples. i) Evaluation of the sensitivity and specificity of SERS‐LFA compared with standard qPCR using a confusion matrix. Data are presented as mean ± s.d. (n = 3).

In order to demonstrate the reliability of the multimodal LFA, *K. pneumoniae* and *S. pneumoniae* (10^5^ CFU/mL) were spiked into normal saliva, urine, and environmental river water samples, for each sample, five individual strips were used. As shown in **Figure** **6**a–c and *K. pneumoniae* and *S. pneumoniae* can be readout by SERS/colorimetric/photothermal signals with high stability (RSD<10%). Moreover, the recovery rate of *K. pneumoniae* and *S. pneumoniae* pathogens (10^3^–10^5^ CFU/mL) in saliva/urine/river water samples were also detected. As shown in Figure S21 and Table S3 (Supporting Information), recovery for *K. pneumoniae* and *S. pneumoniae* ranged from 86.5% to 110.8% and 91.7% to 118.4% in saliva/urine/river water samples. These results indicate that the multimodal LFA platform can be used for dual pathogens detection in biological samples (saliva, urine) and environmental samples (river water) with good stability and feasibility. Subsequently, the proposed LFA was employed for clinical saliva specimens from patients with *S. pneumoniae* infection. A total of ten *S. pneumoniae*‐positive saliva specimens (S1–S10) were collected for LFA detection, five healthy saliva specimens (H1‐H5) were also used as a control. As shown in Figure 6d and Figure S22a (Supporting Information), before colorimetric catalysis, S3, S4, S6, and S7 specimens cannot be visually detected (T2 line), while after colorimetric catalysis, all of ten specimens can be visually detected. The correlation analysis by using of colorimetric catalysis‐LFA and standard qPCR method was performed and shown in Figure 6e, it can be seen that the Pearson correlation coefficient (r) was ‐0.96, indicating the high consistency between of our colorimetric‐catalytic LFA and the qPCR result. To verify the multimode LFA, SERS signals on the T2 lines of clinic specimen strips before colorimetric catalysis (shown in Figure S15, Supporting Information) were also detected and compared with qPCR result, as shown in Figure 6g,h, the ten positive samples showed much higher SERS signals than that of negative controls, for correlation analysis, the Pearson correlation coefficient (r) was ‐0.97, confirming the high consistency between of SERS mode LFA and the qPCR method. The confusion matrix of the colorimetric and the SERS mode with qPCR in Figure 6f,i indicated the 100% accuracy by using our multimode LFA in clinic *S. pneumoniae*‐positive samples recognition. Notably, qPCR requires complex nucleic acid amplification steps, expensive equipment, and skilled technicians which restricts its application in resource‐limited settings, whereas, our multimode LFA can recognize *S. pneumoniae*‐positive samples within 15 min without any pretreatment with high accuracy, the integrating optional three‐way signal output mode, which is comparable to existing multi‐mode POCT sensors^[^ ^59^, ^60^ ^]^ and can be applied in multiple scenarios of POCT screening.

## 3 Conclusion

In summary, we have developed a ligand‐driven annular‐epitaxial growth method to obtain CuS‐Au heterostructures, which indicated superior SERS/POD/ photothermal activities. Based on CuS‐Au assemble LFA, three‐mode platforms can be achieved for visual colorimetric screening and SERS/photothermal sensitive quantitative analysis of the *S. pneumoniae* and *K. pneumoniae* simultaneously within 15 min, the sensitivity was superior or comparable to previous reported LFA platforms for pathogenic bacteria, moreover, to our best knowledge, this is the first rapid method reported for *S. pneumoniae* and *K. pneumoniae* simultaneous recognition. The performance of our three‐mode LFA was validated by using ten positive clinical salivary samples and 100% concordance with the results of qPCR can be obtained. Our research explores a new avenue to obtain well‐defined SERS‐nanozyme CuS‐Au heterodimers and sheds light on the advantages of the three‐mode LFA with improving detection accuracy and sensing range for multiple pathogenic bacterial recognition in clinic samples. Moreover, our assay can be explored for various biomarkers rapid and sensitive recognition and can be a promising tool for disease screening and food safety inspection in various scenarios.

## 4 Experimental Section

### Assembly of the LFA Strip

The LFA was assembled from a previously reported paper.^[^ ^28^ ^]^ In simple terms, two detection lines (T line) and one control line (C line) are modified on the nitrocellulose (NC) film. Modify *K. pneumoniae* antibody on T1 line, *S. pneumoniae* antibody on T2 line, and SA on C line to verify the feasibility of the test strip. The antibodies were coated on NC film with Biodot Xyz5050 sprayer at a rate of 1 µL cm^−1^. The sample pad (glass fiber, GF‐08), the NC member, and the absorption pad (absorption filter paper) were then assembled in a 3 mm intersecting sequence. The assembled strips were then dried at 37 °C for 3h. For testing, the assembled cards are cut into individual strips with a width of 3 mm and stored in a drying cabinet for future use.

### Synthesis of CuS Nanodiscs

CuS nanodiscs were synthesized by a previously reported one‐pot method, and simple modifications were made.^[^ ^38^ ^]^ In short, 16 mg CuCl_2_·2H_2_O and 5 g PVP were added to 70 mL of pure water, 1 mL 20% (NH_4_)_2_S was added to the stirring, and heated to 180 °C for 12h. After cooling to room temperature, centrifuge at 9000 rpm for 7 min and wash three times with ultra‐pure water.

### Synthesis of CuS@ligand@Au

First, 1 mm of CTAC/CTAB or 5 mg of PVP was added to 1 mL CuS solution, and shaken at 600 rpm for 30 min to make the reaction full. Then 5 µL of 1% HAuCl_4_ and 100 mM AA were added and heated to 80 °C for 30 min. Finally, the solution was washed at 8500 rpm/7 min for three times, and the CuS@ligand@Au seed was successfully synthesized. Next, 1 mL CuS@ligand@Au seed was added into 9 mL pure water containing 30 mg PVP and 10 mg hydroxylamine hydrochloride for 15 min in ultrasound reaction. After that, 40 µL of 1% HAuCl_4_ was added for 15 min in ultrasound reaction to produce CuS@ligand@Au. The obtained CuS@ligand@Au solution was washed three times with pure water at 7500 rpm/7 min, and then resuspended with pure water.

### Preparation of the ConA‐CuS@CTAC@Au@4‐MBN Tags

First, 10 mm 4‐MBN solution was added to CuS@CTAC@Au solution, stirred for 4 h, washed three times at 7500 rpm/7 min, then 2 mL of the mixed solution was resuspended with 200 µL 2 mm PB, added 10 µg Biotinylated‐ConA, shook at 600 rpm for 2 h. Finally, 100 µL 10% BSA was added for 1 h oscillation, and centrifugation at 6500 rpm/7 min to complete the preparation of ConA‐CuS@CTAC@Au@4‐MBN tags.

### Preparation of Bacterial Samples and Calculation of Bacterial Capture Efficiency

The bacteria were quantified by classical plate counting, and the strains were cultured on an AGAR plate and then placed in an incubator (37 °C) for 24 h. The colonies were picked from the plate and then resuspended in the PBS buffer. Spread 100 µL bacterial solution evenly on AGAR plate. After 24 h in the incubator, the concentration of the bacterial solution was estimated by the OD value at 600 nm (1 OD ≈ 1 × 10^8^). The bacterial solution was centrifuged at 5000 rpm for 5 min and washed twice with PBS. Finally, according to the calculation results, the bacterial solution was diluted to the required concentration. The capture efficiency was calculated by mixing a solution containing *K. pneumoniae*/*S. pneumoniae* with 10 µL ConA‐Fe_3_O_4_/WGA‐Fe_3_O_4_ (10 mg mL^−1^). After incubating for 40 min, tags‐pathogen will be magnetically adsorbed. Subsequently, the supernatant was evenly coated on an AGAR plate and cultured at 37 °C for 24 h. The capture efficiency is equal to 1 minus the ratio of remaining bacteria in the supernatant after culture to the blank control group.

### SERS/Catalytic Colorimetric/Photothermal Multimode Detection of *K. pneumoniae*/*S. pneumoniae* Based on LFA

First, ConA‐CuS@CTAC@Au@4‐MBN (0.1 mg mL^−1^, 2 µL) was incubated with a series of concentrations of *K. pneumoniae*/*S. pneumoniae* (0–3.75 × 10^6^ CFU/mL, 5 µL) for 10 min, and then the sample was mixed with 70 µL running buffer (including PBS, 1%Tween20 and 1.5% milk). The mixture sample was loaded onto the test strip for Immunochromatographic detection. After reaction for 30 min, amplified SERS/ Catalytic colorimetric/photothermal signals were detected respectively. For SERS detection, Raman spectrometer was used to read SERS signals on the T line. 30 points were randomly measured on each test line, and the average value was calculated to ensure the accuracy and stability of SERS result. For colorimetric signals, 1 µL acetic acid buffer containing 3 mm TMB and 6 mm H_2_O_2_ drops was added to the T line for colorimetric development. Smartphone and ImageJ software were used to analyze the intensity of the gray value of the corresponding region on the T‐line. For the photothermal signal, 808 nm laser with a power density of 1.5 W cm^−2^ was used to illuminate the T line for 1 min for temperature detection, and then a handheld thermal infrared imager was used to record the thermal image and corresponding temperature. In order to reduce the experimental error, each sample was tested separately three times, and the average value of the data was taken.

### Clinical Sample Detection and Recovery Rate Analysis

To evaluate the feasibility of the ConA‐CuS@CTAC@Au@4‐MBN LFA platform for *K. pneumoniae*/*S. pneumoniae* detection, saliva, urine, and river water were used as biological matrix for recovery analysis. First, the standard *K. pneumoniae*/*S. pneumoniae* solution were added into the above three biological matrix, and then examined by the ConA‐CuS@CTAC@Au@4‐MBN assemble LFA platform. The LFA testing procedure for clinical samples was consistent with the above procedure and 10 µL of collected saliva samples were added for each testing. In order to perform qPCR detection of the clinical samples, nucleic acid release solution was used to react with saliva samples for 5 min to release nucleic acid, and then qPCR kit (TIANGEN) was used for detection.

### Statistical Methods

All experiments were performed in three replicates (n = 3) unless otherwise noted. Numerical data are expressed as mean ± standard deviation (SD). The sizes of Au seeds were analyzed by using the software Nano Measurer 1.2 to calculate the mean and standard deviation of Au seeds in the TEM images. Consistency analysis between LFA and qPCR clinical data was performed using GraphPad Prism 9.5 software by calculating the Pearson correlation coefficient (r). The interpretation criteria for the correlation coefficient were as follows: |r| ≥ 0.8 indicates a strong correlation, 0.5 ≤ |r| < 0.8 indicates a moderate correlation, and |r| < 0.5 indicates a weak correlation.

## Conflict of Interest

The authors declare no conflict of interest.

## Supporting information



Supporting Information

## Data Availability

The data that support the findings of this study are available in the supplementary material of this article.
