# Supplementary material for: Ligand‐Driven Annular‐Epitaxial Growth of CuS‐Au Heterostructures as Trinity Plasmonic Nanozyme for Multimode Diagnosis of Pathogenic Bacteria
Source: Adv Sci (Weinh). 2025 Apr 24;12(23):2500134. doi: 10.1002/advs.202500134 (PMC12199314; doi:10.1002/advs.202500134)
Supplement: Supplementary file 1 — Supporting Information [file ADVS-12-2500134-s001.docx]

Supporting Information

**Ligand-Driven** **Annular-Epitaxial Growth of** **CuS-Au** **Heterostructures as** **Trinity Plasmonic Nanozyme for Multimode Diagnosis of** **Pathogenic Bacteria**

*Xiaorui Lin, Pengyou Zhou, Miao An, Chenyi Zhu, Yuanfeng Pang*, Rui Xiao**

Capital Medical University, No.10 Xitoutiao, You An Men, Beijing 100069, China

State Key Laboratory of Pathogen and Biosecurity, Academy of Military Medical Sciences, Beijing 10071, China

E-mail: yuanfengpang@ccmu.edu.cn; ruixiao203@163.com

**Content**

Experimental Section 3

Tables 6

Figures 15

References 16

# Experimental Section

## Reagents

Copper (II) chloride dihydrate (CuCl_2_·2H_2_O), Ammonium sulfide solution ((NH_4_)_2_S), HAuCl_4_·4H_2_O, 1×PBS (pH=7.4), Polyvinylpyrrolidone (PVP), Hexadecyltrimethylammonium bromide (CTAB), Hexadecyltrimethylammonium chloride (CTAC), L-ascorbic acid (AA), Streptavidin (SA) and NH_2_OH·HCl were purchased from Sigma-Aldrich (USA). Wheat Germ Agglutinin (WGA)-Biotinylated (B-1025-5) and Concanavalin A (ConA)-Biotinylated (B-1005-5) were purchased from Vector Laboratories. Anti-*Klebsiella. pneumoniae* (ACT-PrAb-KLEB.P-001) and Anti-*Streptococcus. pneumonia* (ACT-rmAb-STREP-002) were purchased from ACTHTEAM, LLC (USA). 4-Mercaptobenzonitrile(4-MBN), Bovine serum albumin (BSA), N-(3- dimethylaminopropyl)-N’-ethyl carbodiimide (EDC), N-hydroxy succinimide (NHS), 2-(N-morpholino) ethane sulfonic acid (MES), Tween-20 were purchased from Sigma-Aldrich (USA). Macroporous nitrocellulose (NC) membrane CN95 and CN140 were purchased from Sartorius (Spain). Nucleic acid release solution（NR216）purchased from GenDx Biotech. Real Universal Color PreMix (SYBR Green) purchased from TIANGEN.

## Apparatus

The UV-vis spectra were collected by a Shimadzu 2600 spectrometer. The zeta potential data of the nanomaterials were detected by Malvern Nano-ZS90 Zetasizer. The structure and morphology were characterized by Transmission electron microscopy (TEM) using a FEI Tecnai G2 F20 S-TWIN TMP transmission electron microscope at 200 kV. All Raman spectra were collected on a XploRA PLUS: MicroRaman Spectrometer. The acquisition time for sample Raman detection was typically 10 s. A 785 nm laser with 10 mW powers was used for SERS detection. To make the SERS results more accurate and reduce experimental errors, the test lines of each LFA were randomly measured 30 times to calculate the average value for data analysis. The photothermal signal was recorded with a FLIR E4 thermal infrared imager (FLIR Systems Inc., USA) under the irradiation of an 808 nm laser (Hi-Tech Optoelectronics Co., Ltd., China). For the colorimetric signals, 1 μL of acetate buffer (containing TMB (1mM) and H_2_O_2_ (2mM)) was smeared on the T line. After 1min, the amplified colorimetric signal was recorded with a smart phone, and the intensity of the gray value of the corresponding area on the T-line was analyzed with ImageJ software. Finally, all data were smoothed and baseline corrected using the bundled software.

## Finite-difference time-domain (FDTD) simulation

Here, we have calculated the electric field distribution of the nanostructures using 3D-FDTD. During the calculations, both x, y and z directions are set to perfectly match the layer conditions to prevent unphysical scattering. In addition, in order to obtain accurate calculation results, we meshed the whole simulation area as 0.15 nm × 0.15 nm × 0.15 nm. importantly, the total field scattering field plane wave was employed as the excitation light source vertically incident to the nanostructures. We confirmed the convergence time as 1 × 10^-5^ fs to ensure the convergence of the computational results. Finally, an electric field monitor was employed to obtain the electric field distribution of the nanostructures.

## Ethical Statement

A total of 10 S. pneumoniae clinical saliva specimens and 5 healthy saliva specimens from the Capital Institute of Pediatrics (Ethical approval number: SHERLL-2024-033). All subjects provided written informed consent.

# Tables

**Table S1** Kinetics parameters of Au-Cu based nanozymes

| Catalyst | Substrate | K_m_  (mM) | V_max_  (M s^-1^) | Refs. |
| --- | --- | --- | --- | --- |
| HRP | H_2_O_2_ | 3.7 | 8.7×10^-8^ | [1] |
| Cu_2_O/Au-Pt@MOF | H_2_O_2_ | 22.4 | 4.1×10^-8^ | [2] |
| AuCuPt | H_2_O_2_ | 71.7 | 1.5×10^-5^ | [3] |
| Au@Cu_2_O | H_2_O_2_ | 29.30 | 1.2×10^-7^ | [4] |
| CuS@Au | H_2_O_2_ | 5.8 | 1.1×10^-7^ | This work |
| CuS@CTAC@Au | H_2_O_2_ | 4.3 | 1.6×10^-7^ | This work |

**Table S2** Rapid amplification-free detection of *K. pneumoniae*/*S. pneumoniae* antigen by using of various LFAs.

| **Detection Method** | ***K.P***  **(LOD CFU/mL)** | | ***S.P***  **(LOD CFU/mL)** | **Number of channels** | **Ref.** |
| --- | --- | --- | --- | --- | --- |
| Fluorescent-LFA | - | 13 | | 1 | [5] |
| Magnetic-Fluorescent-LFA | - | 10 | | 1 | [6] |
| SERS-LFA | - | 46 | | 1 | [7] |
| Fluorescent microsphere-LFA | - | 10^3^ | | 1 | [8] |
| Electrochemical-LFA | - | 10 | | 1 | [9] |
| Colloidal gold detection | 1.2×10^5^ | 1.3×10^5^ | | 1 | This work |
| SERS mode | 2.0 | 2.0 | | 2 | This work |
| Photothermal mode | 1.8×10^2^ | 3.6×10^2^ | | 2 | This work |
| Nanozyme colorimetric mode | 2.9×10^2^ | 6.5×10^2^ | | 2 | This work |

**Table S3** Recoveries of *K. pneumoniae* or *S. pneumoniae* at different concentrations in different samples.

| **Samples`** | **Species** | **Spiked**  **(CFU/mL)** | **Detected**  **(CFU/mL)** | **RSD**  **(%, n=3)** | **Recovery**  **(%, n=3)** |
| --- | --- | --- | --- | --- | --- |
| Saliva | *K. pneumoniae* | 1.5 × 10^5^ | 1.6 × 10^5^ | 0.5 | 103.4 |
|  |  | 3.0 × 10^4^ | 3.0 × 10^4^ | 4.1 | 99.9 |
|  |  | 6.0 × 10^3^ | 6.1 × 10^3^ | 2.9 | 101.4 |
|  | *S. pneumoniae* | 1.5 × 10^5^ | 1.5 × 10^5^ | 2.5 | 103.0 |
|  |  | 3.0 × 10^4^ | 2.9 × 10^4^ | 2.4 | 98.3 |
|  |  | 6.0 × 10^3^ | 6.0 × 10^3^ | 6.1 | 100.1 |
| Urine | *K. pneumoniae* | 1.5 × 10^5^ | 1.4 × 10^5^ | 1.6 | 94.3 |
|  |  | 3.0 × 10^4^ | 3.3 × 10^4^ | 3.2 | 110.8 |
|  |  | 6.0 × 10^3^ | 6.3 × 10^3^ | 2.5 | 105.4 |
|  | *S. pneumoniae* | 1.5 × 10^5^ | 1.4 × 10^5^ | 1.2 | 93.0 |
|  |  | 3.0 × 10^4^ | 3.1 × 10^4^ | 1.5 | 103.5 |
|  |  | 6.0 × 10^3^ | 6.3 × 10^3^ | 3.5 | 104.2 |
| River water | *K. pneumoniae* | 1.5 × 10^5^ | 1.3 × 10^5^ | 0.2 | 86.5 |
|  |  | 3.0 × 10^4^ | 2.9 × 10^4^ | 0.9 | 97.2 |
|  |  | 6.0 × 10^3^ | 5.8 × 10^3^ | 8.4 | 97.2 |
|  | *S. pneumoniae* | 1.5 × 10^5^ | 1.4 × 10^5^ | 4.5 | 91.7 |
|  |  | 3.0 × 10^4^ | 3.0 × 10^4^ | 0.8 | 100.5 |
|  |  | 6.0 × 10^3^ | 7.1 × 10^3^ | 4.6 | 118.4 |

# Figures

**
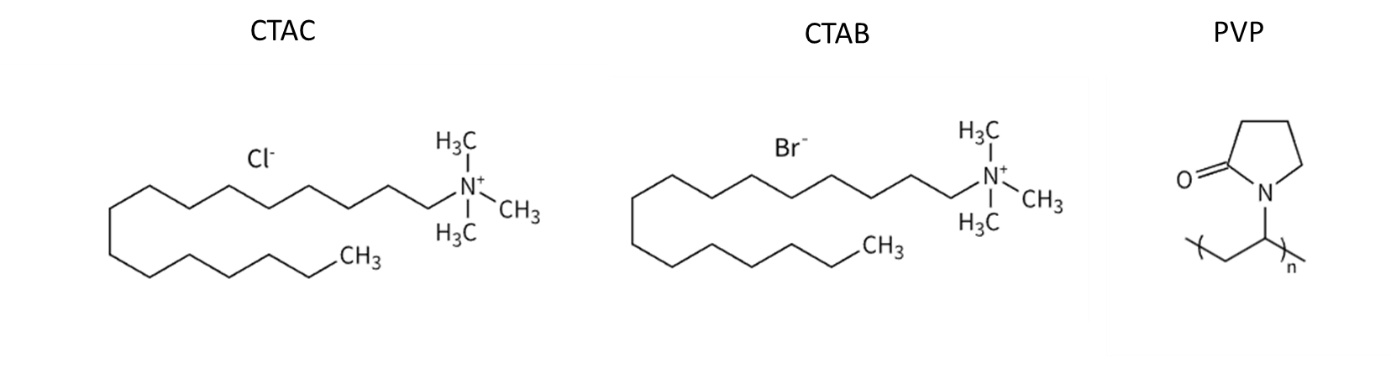
**

**Figure S1.** Structures of the ligands used to synthesize CuS@Au nanozymes.

**
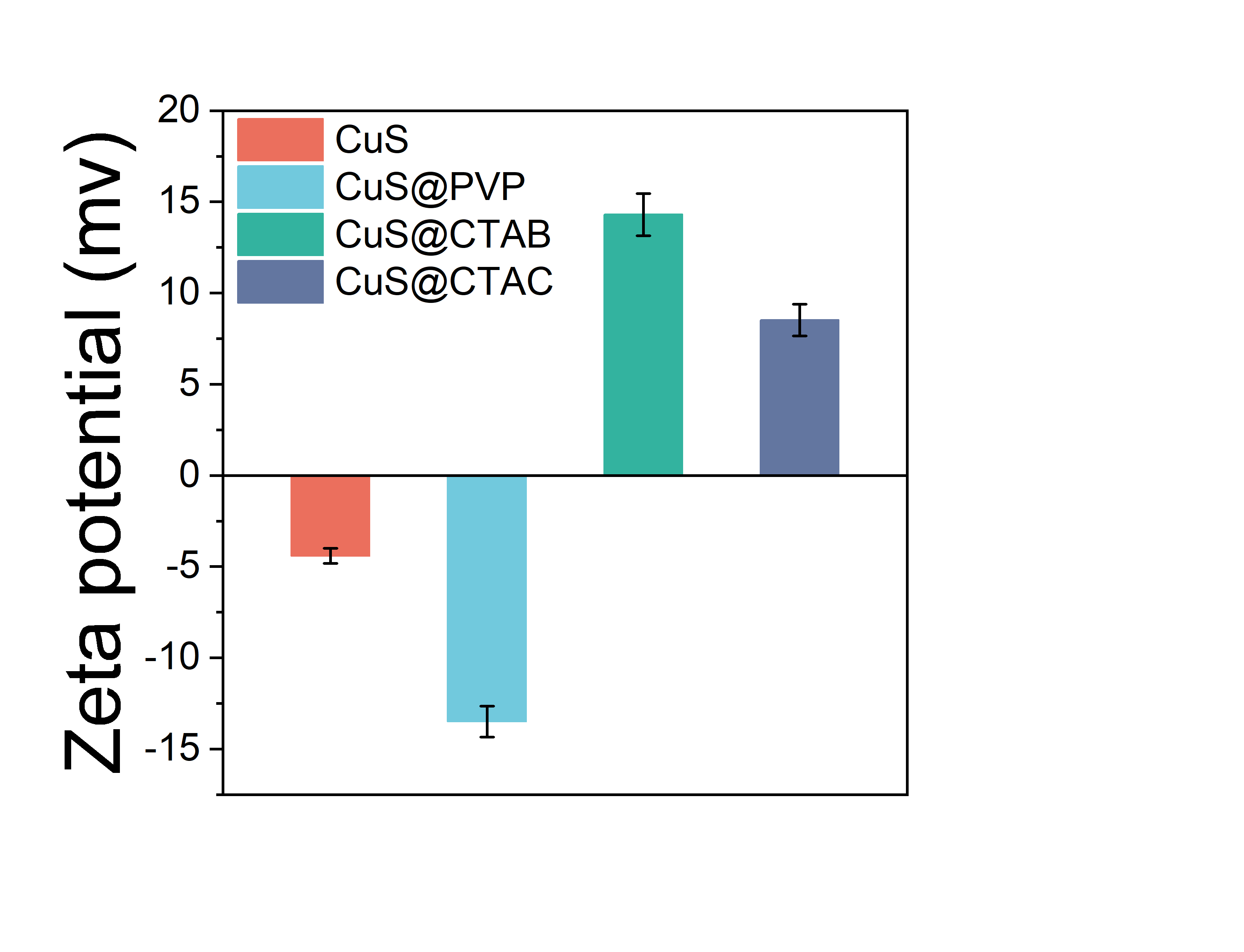
**

**Figure S2.** The zeta potential of CuS, CuS@PVP, CuS@CTAB, and CuS@CTAC (n=3).


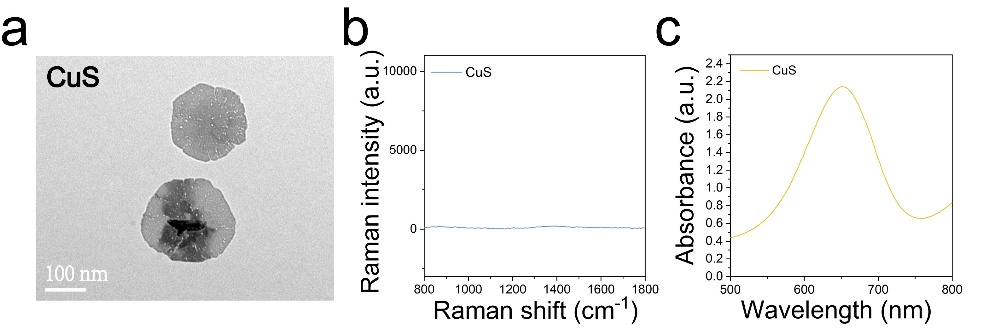


**Figure S3**. Characteristic of CuS. (a)TEM image, (b) SERS spectrum and (c) POD-mimic activity.


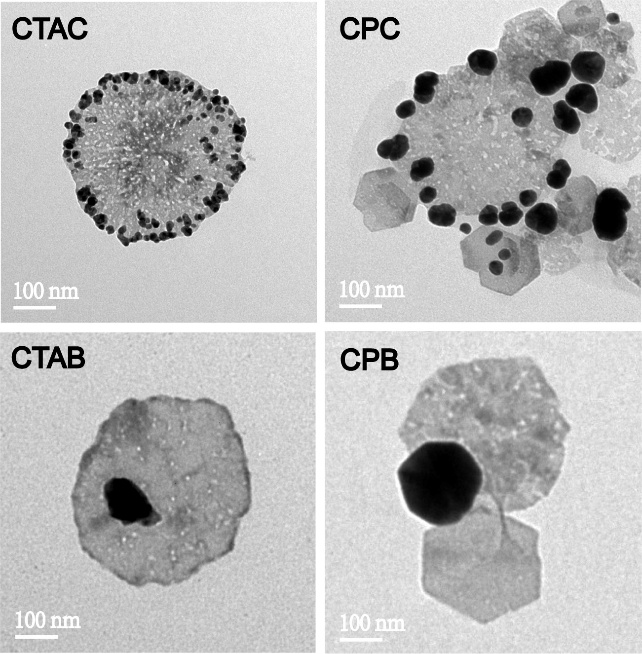


**Figure S4.** TEM images of Au seed growing on the CuS substrates regulated by different ligands (CTAC, CPC, CTAB, CPB).


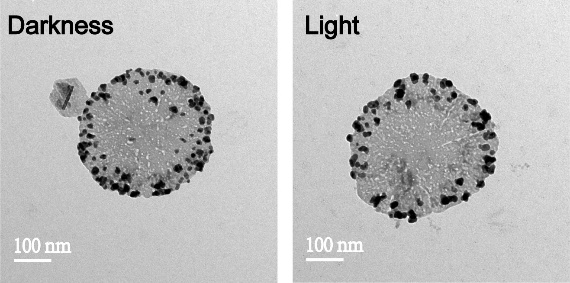


**Figure S5.** TEM images of Au seed growth on CTAC modified CuS in ambient light and dark conditions with same other conditions.

**
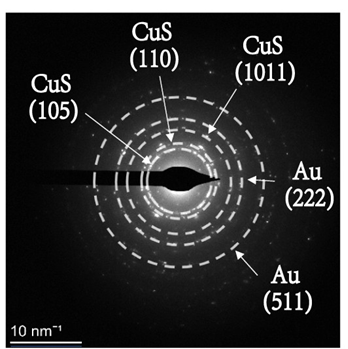
**

**Figure S6.** Selected area electron diffraction (SAED) pattern of CuS@CTAC@Au.

**
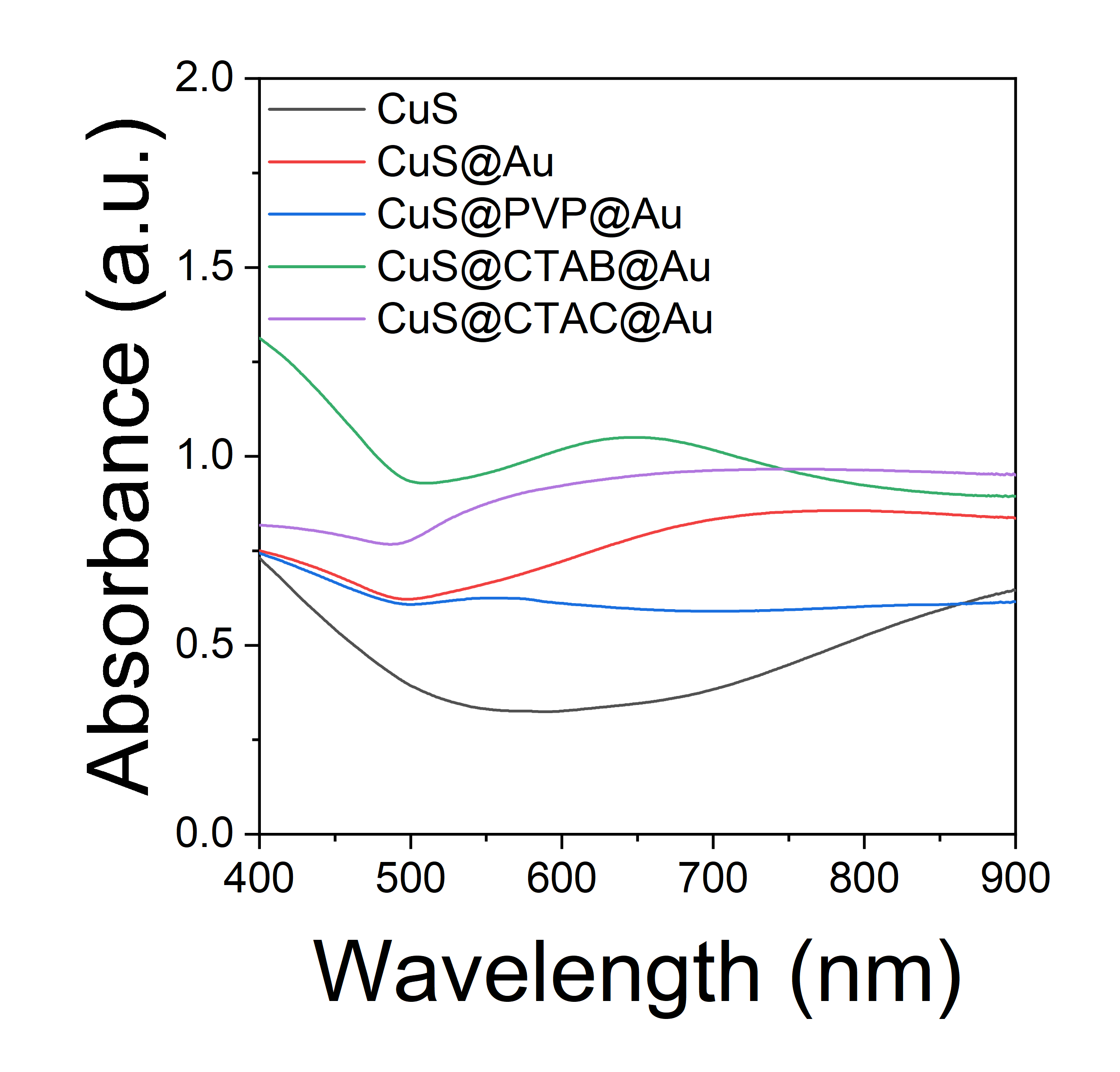
**

**Figure S7.** The UV−vis absorption spectra of CuS@Au with different ligands.


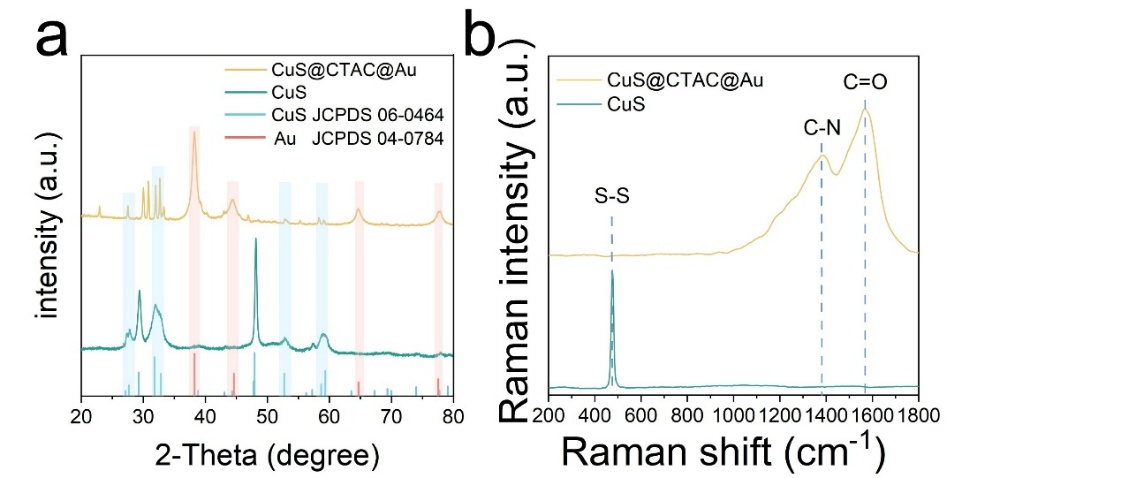


**Figure S8.** XRD(a) and Raman(b) spectra of CuS nanodisc and CuS@CTAC@Au.


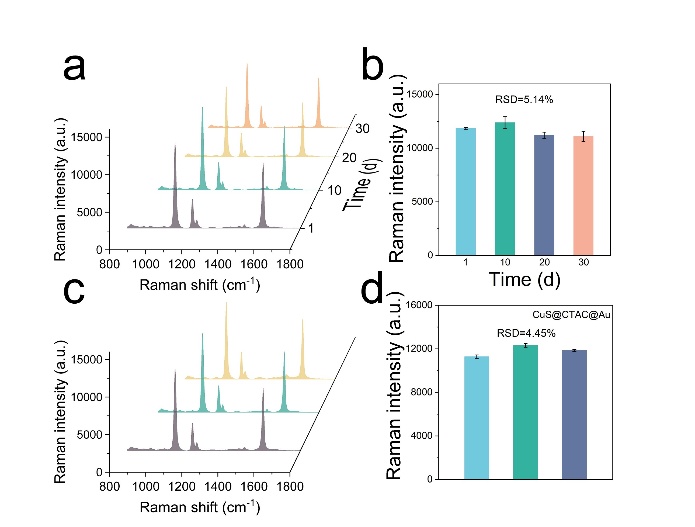


**Figure S9.** (a-b) SERS spectrum of CuS@CTAC@Au@4-MBN at different time periods and SERS intensity corresponding to 1074 cm^−1^ (characteristic peak of 4-MBN). (c-d) the corresponding SERS intensity (n=3).


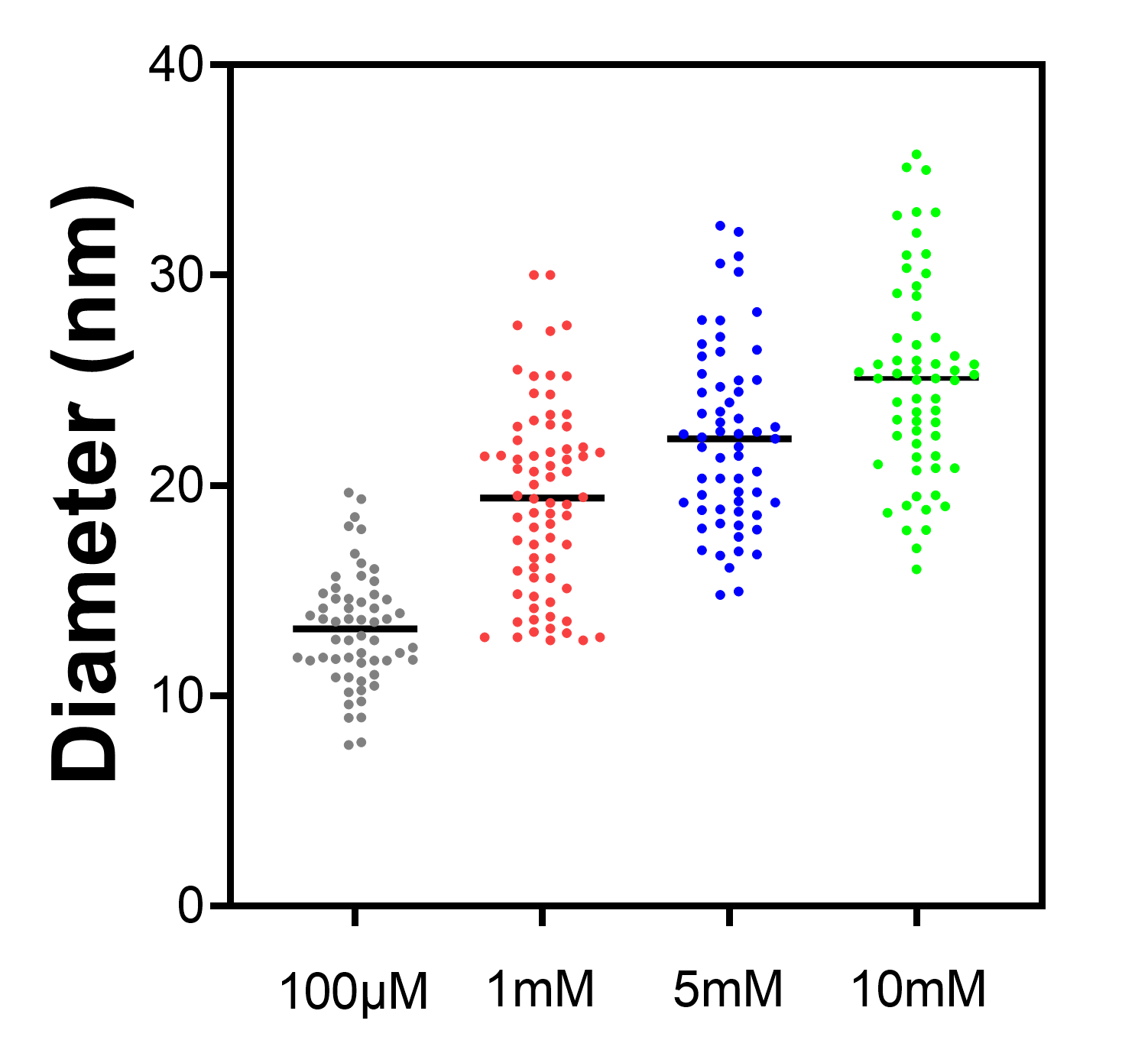


**Figure S10.** The Au seed average particle size of CuS@CTAC@Au seed at different CTAC concentrations.


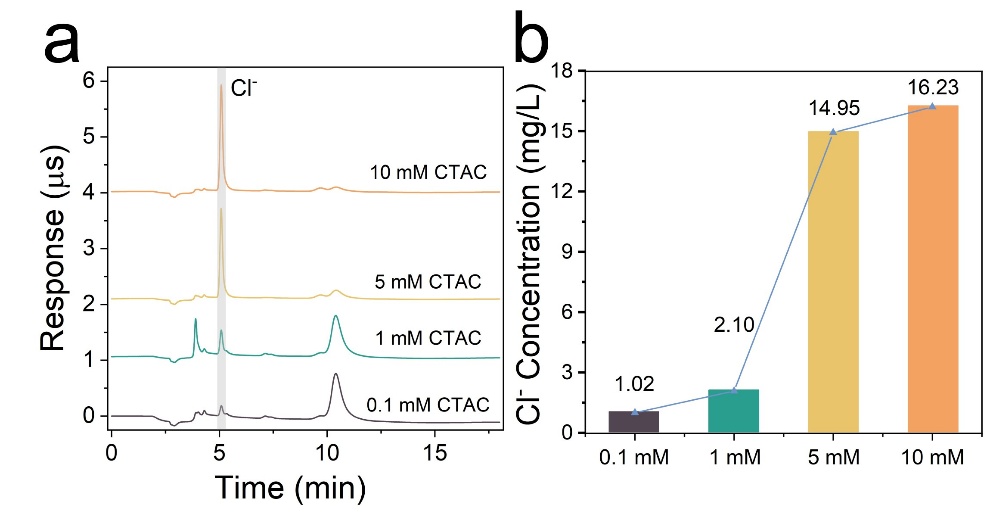


**Figure S11.** (a) Cl⁻-quantify ion chromatography of CTAC adsorption on the CuS after modification by different concentrations of CTAC, (b) the corresponding Cl^-^ concentrations in Figure S11a.


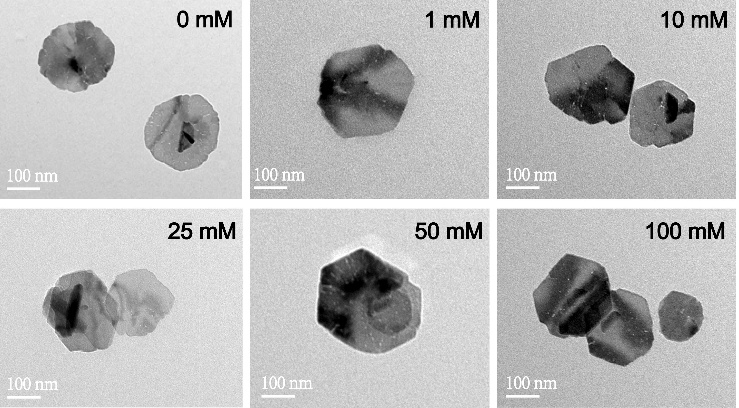


**Figure S12.** TEM images of CuS after modification with different CTAC concentrations.

**
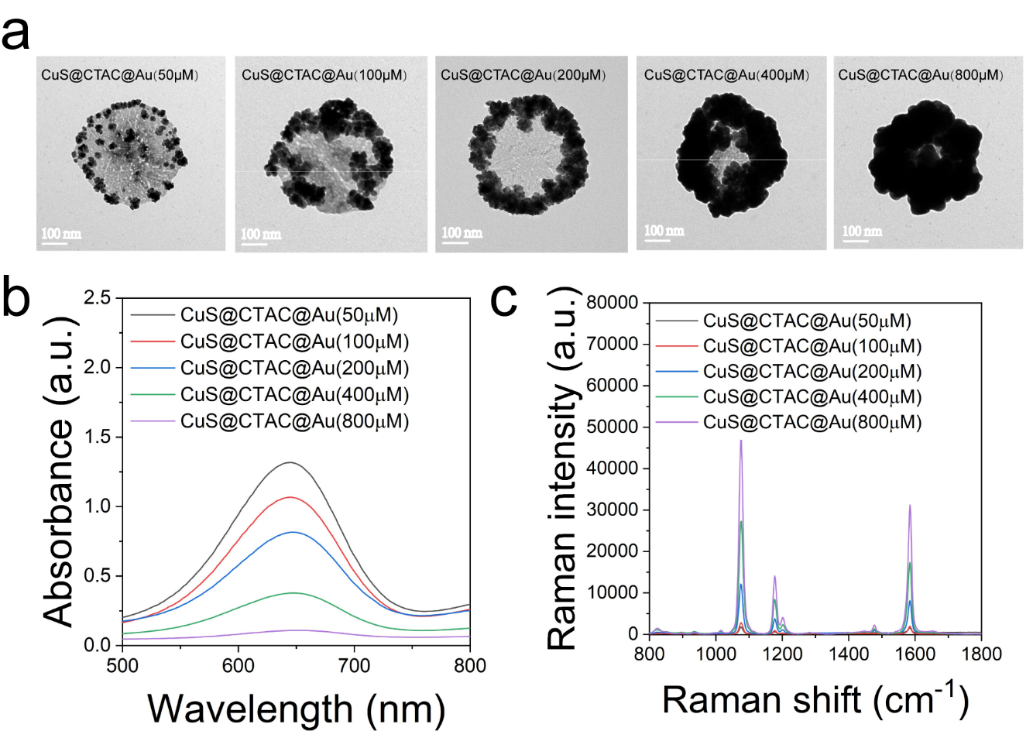
**

**Figure S13.** The TEM images(a), POD-mimic catalytic activity (b), and SERS activity(c) of CuS@CTAC@Au at different HAuCl_4_ additions.

**
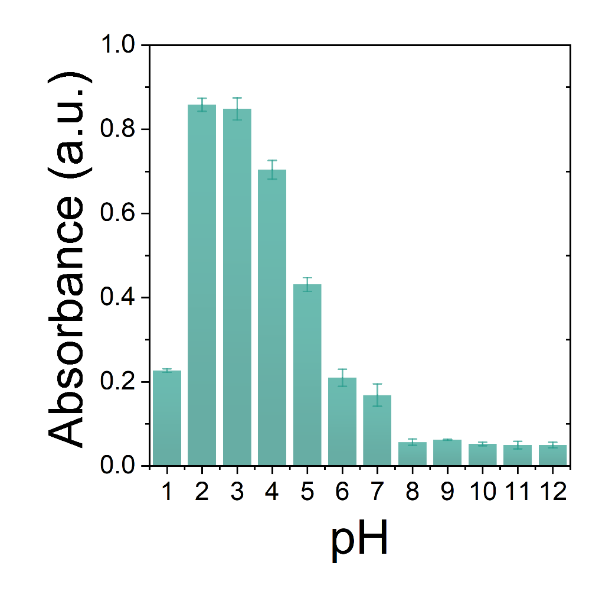
**

**Figure S14.** The POD-mimc catalytic activity of CuS@CTAC@Au nanozymes in pH1~12.


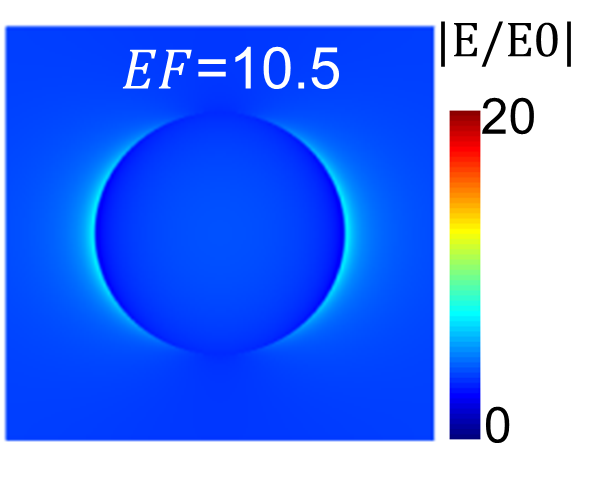


**Figure S15.** FDTD simulations of CuS nanodisc electromagnetic fields.


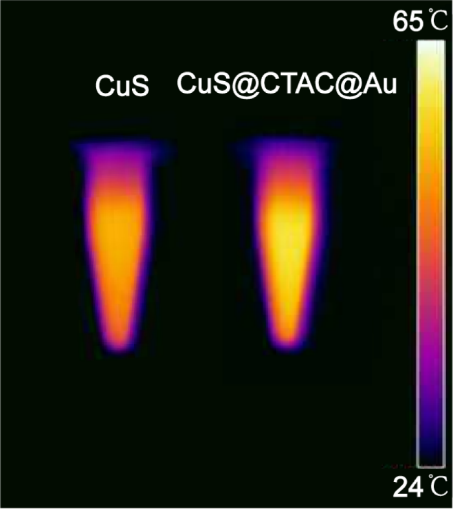


**Figure S16.** Photothermal images of CuS and CuS@CTAC@Au under 808 nm laser irradiation (1.5W /cm^−2^).


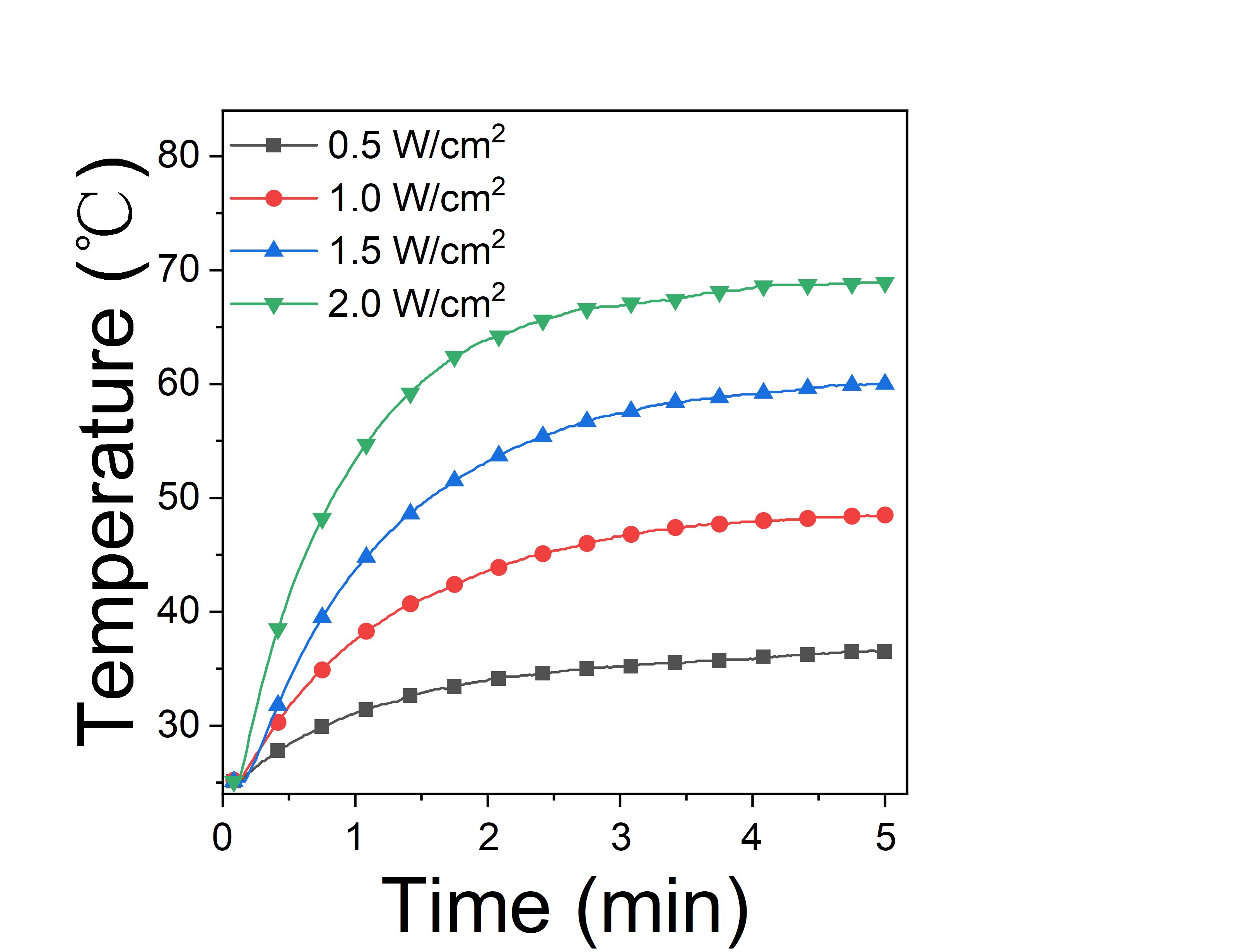


**Figure S17.** Temperature curves of CuS@CTAC@Au with different laser power densities under 808nm laser irradiation.


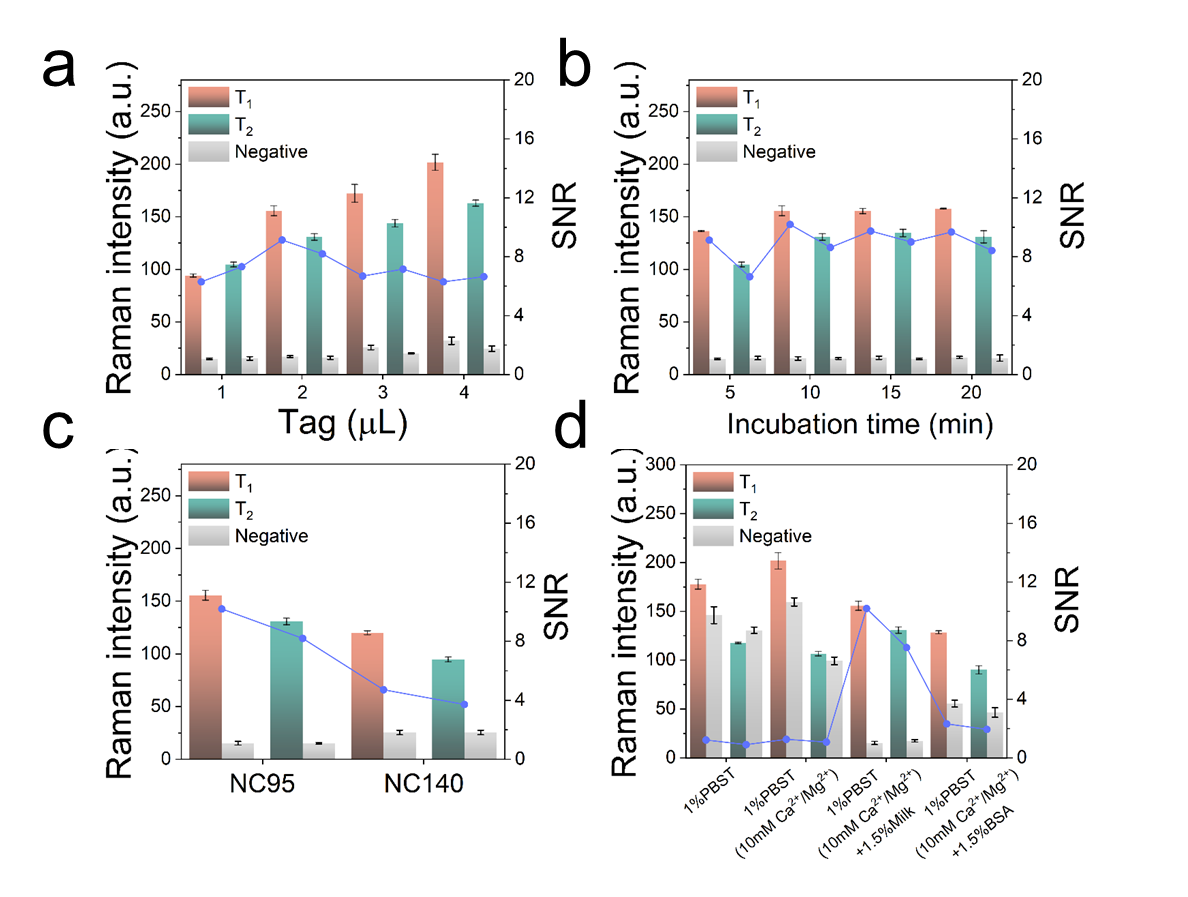


**Figure S18.** Optimization of experimental conditions for targeted pathogens detection. PBS was used as a negative control. (a) Amount of SERS tags (b) incubation time(c) kinds of nitrocellulose filter membrane (d) running buffer. Data was presented as mean ± s.d. (n = 3 independent experiments).


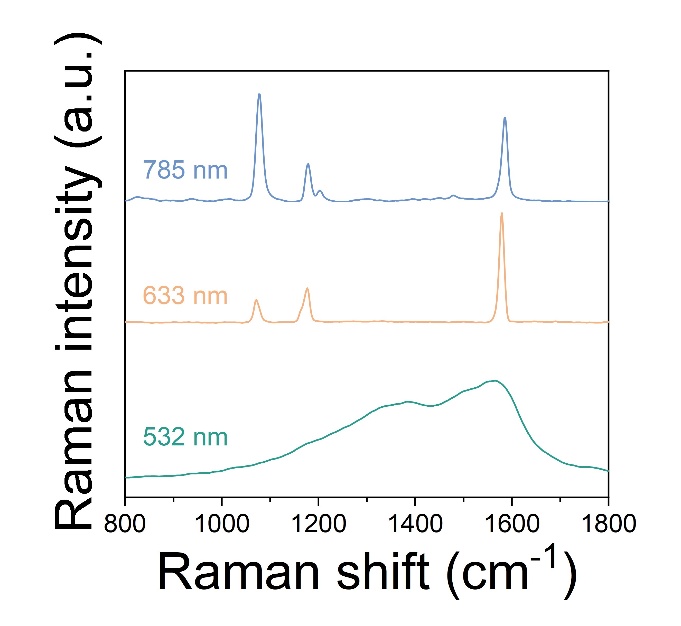


**Figure S19.** The corresponding SERS spectra of CuS@CTAC@Au@4-MBN collected on the T line irradiated by different excitation lasers.

**
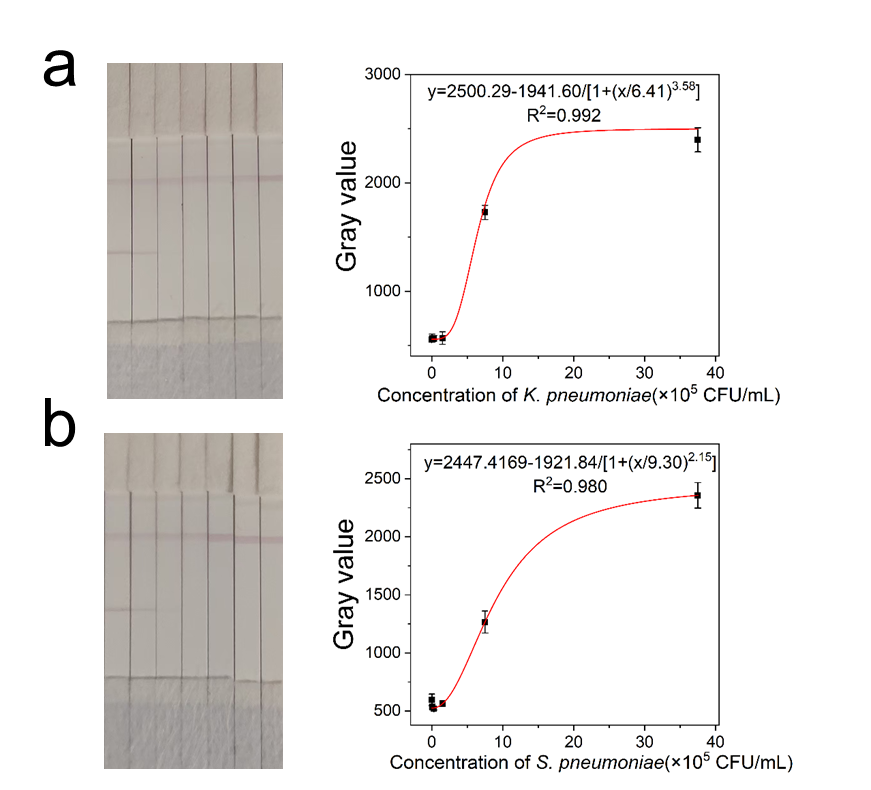
**

**Figure S20.** The pictures and the test line measured by image J of the *K. pneumoniae*-AuNP LFA strips (a) and the *S. pneumoniae*-AuNP LFA strips (b) . Data was presented as mean ± s.d. (n = 3 independent experiments).


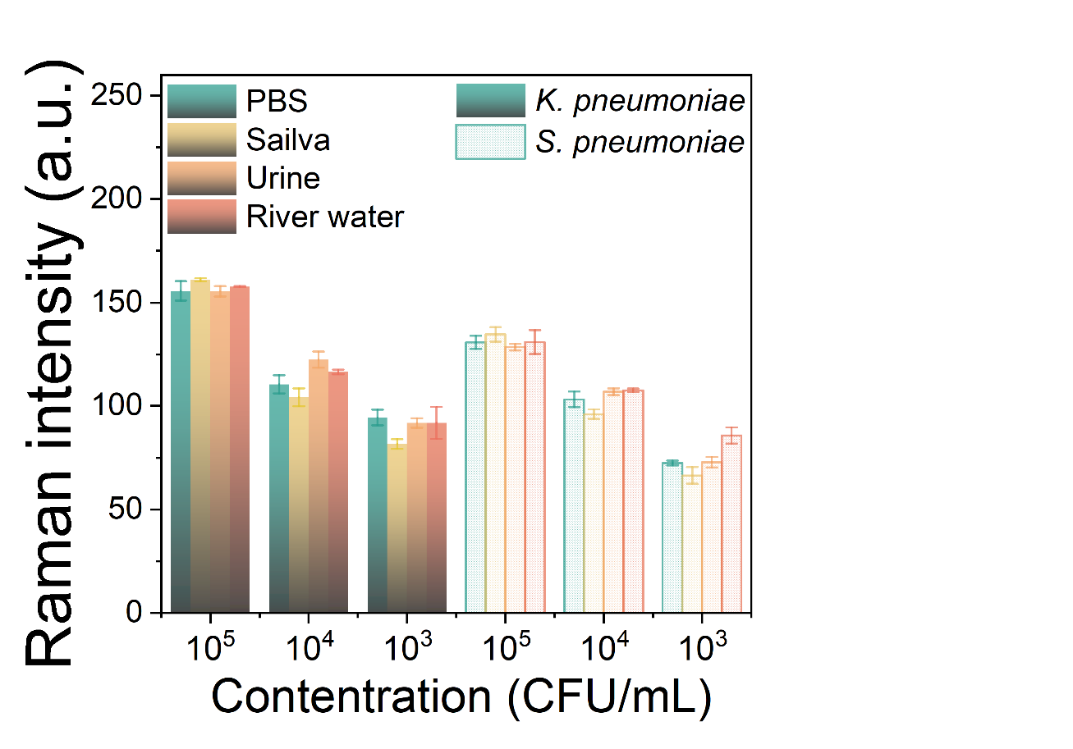


**Figure S21.** The recoveries of different concentrations of pathogens spiked in saliva, urine and river water respectively.


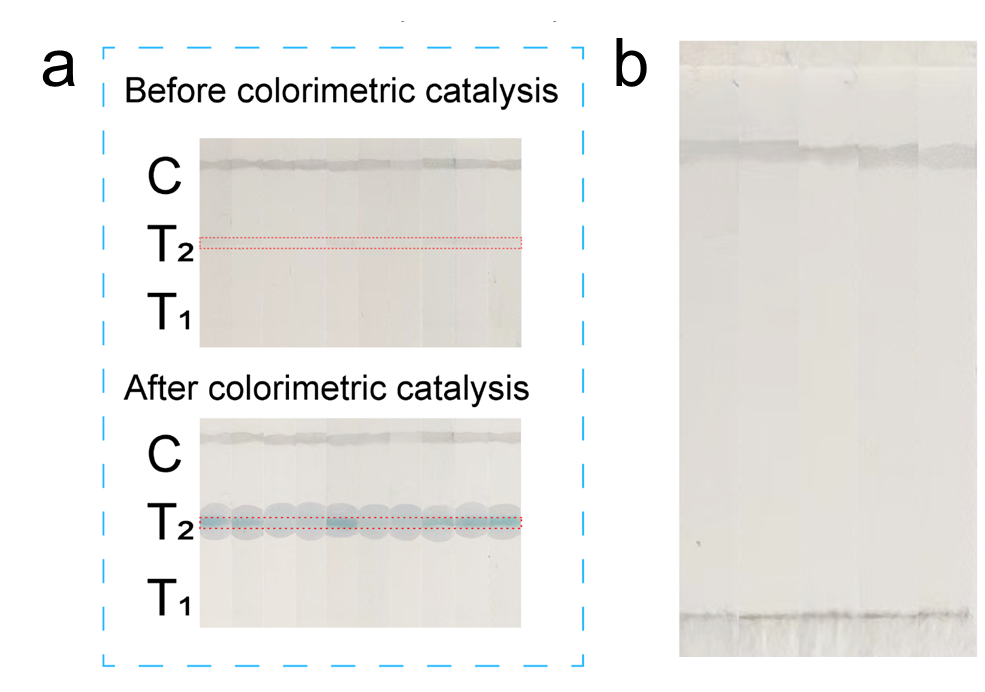


**Figure S22.** (a)Photographs of the strips responding to different clinical saliva samples before and after catalytic amplification. (b)LFA Photographs of 5 healthy saliva specimens(H1~H5).

# References

1. L. Z. Gao, J. Zhuang, L. Nie, J. B. Zhang, Y. Zhang, N. Gu, T. H. Wang, J. Feng, D. L. Yang, S. Perrett, X. Yan. *Nat. Nanotechnol* 2007, 2, 577.
2. Y. Cheng, Y. Xia, Y. Sun, Y. Wang, X. Yin. *Adv. Mater* 2024, 36, 2308033.
3. J. Liu, S. Dong, S. Gai, Y. Dong, B. Liu, Z. Zhao, Y. Xie, L. Feng, P. Yang, J. Lin, *ACS Nano* 2023, 17(20), 20402–20423.
4. J. Zeng, C. Ding, L. Chen, B. Yang, M. Li, X. Wang, F. Su, C. Liu, Y. Huang, *ACS Appl. Mater. Interfaces* 2023, 15, 1, 378–390
5. X. Cheng, S. Zheng, W. Wang, H. Han, X. Yang, W. Shen, C. Wang, S. Wang, *Chemical* *Engineering Journal* 2021, 426.
6. C. Wang, W. Shen, Z. Rong, X. Liu, B. Gu, R. Xiao, S. Wang, *Nanoscale* 2020, 12, 795-807.
7. W. Shen, J. Li, B. Jiang, Y. Nie, Y. Pang, C. Wang, R. Xiao, R. Hao, *Pathogens* 2023, 12.
8. J.Liang, L. Wu, Y. Tang et al, *SENSOR ACTUAT B-CHEM* 2023, 389, 133875.
9. Kunpatee. K, Khantasup. K, Komolpis. K, Yakoh. A, Nuanualsuwan. S, Sain. MM, Chaiyo. S, *Biosens Bioelectron.* 2023, 242, 115742.
